# Soluble expression, purification and characterization of the full length IS*2 *Transposase

**DOI:** 10.1186/1759-8753-2-14

**Published:** 2011-10-27

**Authors:** Leslie A Lewis, Mekbib Astatke, Peter T Umekubo, Shaheen Alvi, Robert Saby, Jehan Afrose

**Affiliations:** 1 Department of Biology, York College of the City University of New York, Jamaica, New York, 11451, USA; 2 Program in Cellular, Molecular and Developmental Biology, Graduate Center, City University of New York, New York, New York 11016, USA; 3 Johns Hopkins University, Applied Physics Laboratory, Laurel, MD 20723, USA; 4 Accera Inc, Broomfield, CO 80021, USA; 5 Ross Medical School, Roseau, Dominica; 6 Department of Occupational Therapy, York College of the City University of New York, Jamaica, New York, 11451, USA; 7 Skirball Institute of Biomolecular Medicine, New York University School of Medicine, New York, New York, 10016, USA

## Abstract

**Background:**

The two-step transposition pathway of insertion sequences of the IS*3 *family, and several other families, involves first the formation of a branched figure-of-eight (F-8) structure by an asymmetric single strand cleavage at one optional donor end and joining to the flanking host DNA near the target end. Its conversion to a double stranded minicircle precedes the second insertional step, where both ends function as donors. In IS*2*, the left end which lacks donor function in Step I acquires it in Step II. The assembly of two intrinsically different protein-DNA complexes in these F-8 generating elements has been intuitively proposed, but a barrier to testing this hypothesis has been the difficulty of isolating a full length, soluble and active transposase that creates fully formed synaptic complexes *in vitro *with protein bound to both binding and catalytic domains of the ends. We address here a solution to expressing, purifying and structurally analyzing such a protein.

**Results:**

A soluble and active IS*2 *transposase derivative with GFP fused to its C-terminus functions as efficiently as the native protein in *in vivo *transposition assays. *In vitro *electrophoretic mobility shift assay data show that the partially purified protein prepared under native conditions binds very efficiently to cognate DNA, utilizing both N- and C-terminal residues. As a precursor to biophysical analyses of these complexes, a fluorescence-based random mutagenesis protocol was developed that enabled a structure-function analysis of the protein with good resolution at the secondary structure level. The results extend previous structure-function work on IS*3 *family transposases, identifying the binding domain as a three helix H + HTH bundle and explaining the function of an atypical leucine zipper-like motif in IS*2*. In addition gain- and loss-of-function mutations in the catalytic active site define its role in regional and global binding and identify functional signatures that are common to the three dimensional catalytic core motif of the retroviral integrase superfamily.

**Conclusions:**

Intractably insoluble transposases, such as the IS*2 *transposase, prepared by solubilization protocols are often refractory to whole protein structure-function studies. The results described here have validated the use of GFP-tagging and fluorescence-based random mutagenesis in overcoming this limitation at the secondary structure level.

## Background

**Figure 1:**
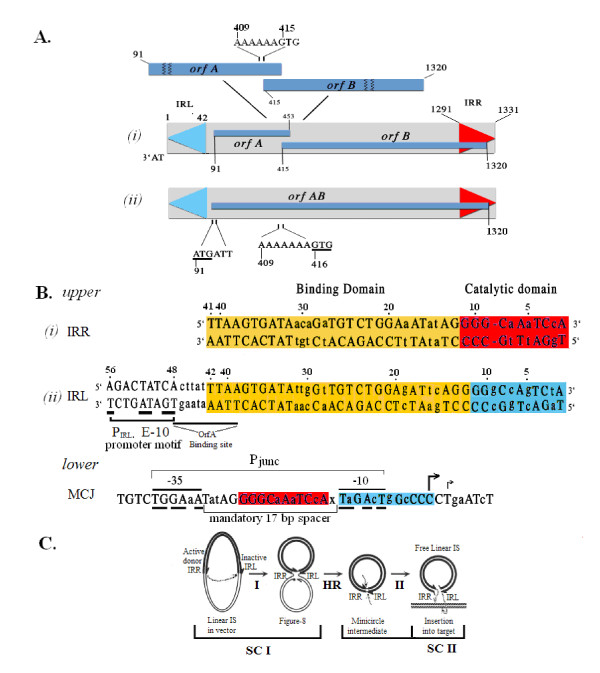
**Organization of the IS*2 *insertion sequence and its transposition pathway**. **(A) **Wild type IS*2 *with left and right inverted repeats (IRL, blue; IRR, red) and the two overlapping open reading frames, *orfA *and *orfB*, expanded to show the detail of the A_6_G slippery codon window which regulates low levels of OrfAB formation (*i*). High levels of the transposase (TPase) are produced by altering the window to A_7_G (*ii*). **(B)***Upper*. Aligned sequences of IRR and IRL ((*i*) and (*ii*)) with the binding domains (yellow) and color coded catalytic domains. Conserved residues are in uppercase and diverged residues are in lower case. The catalytic domain (CD) of IRL contains an additional G/C base pair that is essential for its role in target function [7]. The E-10 promoter, P_IRL_, [19] (*ii*) drives the events of Step I of the transposition pathway [6] resulting in the formation of the minicircle shown in panel C. *Lower*: Abutted ends at the minicircle junction (MCJ), form a more powerful promoter (P_junc_) which indispensably controls the events in Step II of the transposition pathway. The only functional form of P_junc _contains a single base pair spacer (x) which creates the mandatory 17 bp spacer. **(C) **The two-step transposition pathway of IS*2*. Step I (I) occurs in the TPase-DNA complex, the synaptic complex I (SC I). Asymmetric single strand cleavage of the active IRR donor is followed by strand transfer to the donor-inactive IRL target end, creating the figure-of-eight structure. Host replication mechanisms (HR) convert it into a covalently closed double stranded circular intermediate [10], the minicircle. In step II (II) a second synaptic complex (SC II) is assembled. Cleavages at the abutted CDs result in two exposed 3'OH groups which carry out transesterification attacks on the target DNA. CD: catalytic domain; E-10: extended-10 promoter; IRR/IRL: right and left inverted repeats; IS: insertion sequence; MCJ: minicircle junction; orf: open reading frame; SC: synaptic complex.

IS*2*, a 1.3 kb transposable element, is a member of the IS*3 *family, the largest and most widespread family of insertion sequences (IS) ([1,2]; see also ISfinder: http://www-is.biotoul.fr/is.html). These insertion sequences are characterized by terminal imperfect inverted repeats, the right (IRR) and left (IRL) ends, that flank an internal protein coding sequence (Figure 1a). The latter is comprised of two -1 frameshifted overlapping open reading frames, OrfA and OrfB (Figure 1a, i) and is regulated in IS*2 *by a weak extended-10 promoter (E-10) promoter (Figure 1b, ii). Within the overlap, a ribosomal slippage window [3,4], characterized in IS*2 *by an A_6_G motif (Figure 1a, i), enables translational frameshifting to create the functional transposase (TPase) at a low frequency (OrfAB) but an A_7_G mutation (Figure 1a, ii) has permitted the production of an engineered frame-fused OrfAB as the principal translation product [5,6]. The ends of these elements are bipartite structures (Figure 1b, *upper*) with internal protein binding domain and outer catalytic domains (CD) [7,8] terminating in most cases with a CA-3' dinucleotide that is the essential substrate for cleavage and joining (donor function) reactions, see [9]. In IS*2*, IRL terminates with a TA-3' dinucleotide which creates a functional Pribnow box for a minicircle junction promoter (see below).

Transposition mechanisms, initially discovered in the IS*3 *family (see [2]) have been described as a two-step copy and paste pathway [10] which is now quite widespread and is found in several other families of insertion sequences, such as IS*30*, IS*21 *and IS*256 *[11-14]. In IS*3 *family members, IS*911 *[8,15] and IS*2 *(Lewis *et al*, Protein-DNA interactions define the mechanistic aspects of circle formation and insertion reactions in IS2 transposition, submitted), Step I occurs within a synaptic complex (SC) or transpososome (Figure 1c, SC I) that is formed when the TPase binds to the two ends. In general, however, in these circle-forming elements the first step involves a circularization process (Figure 1c) in which either end (optionally) is the substrate for an asymmetric cleavage reaction that leads to a donor-to-target intrastrand joining reaction near the other end to form a branched figure-of-eight (F-8) structure [6,16-18] Host replication mechanisms [10] convert the F-8 into a covalently closed double stranded minicircle (Figure 1c, HR) with the abutted ends generally separated by one or more base pairs derived from the host DNA flanking the target end. These abutted ends constitute the minicircle junction (MCJ) at which a powerful promoter (Figure 1b, *lower*; P_junc _[19-21]) is assembled and generates the higher levels of TPase needed for the formation of the second synaptic complex (Figure 1c, SC II).

In SC II, the MCJ, a reactive junction, is the substrate for strand transfer reactions; it is cleaved at the abutted termini of IRR and IRL, creating 3'OH groups which permit both ends to function symmetrically as donors (Figure 1c, Step II). Thus it has been proposed that intrinsically different transpososomes must be assembled at each of the two steps [7,8]. This is particularly true for IS*2*. Although both right and left ends in other IS*3 *family elements, such as IS*911 *[16], IS*3 *[22] and IS*150 *[23], possess donor function in Step I reactions, in IS*2 *the right end is the exclusive donor and the left end the only functional target; this type of asymmetry has also been described for copies of IS*256 *in Tn*4001 *[13]. In IS*2*, the left end has evolved through altered residues at positions 2 (creating a TA-3' terminal dinucleotide), 5 and 7 and an additional base pair at position 9 in its catalytic domain (Figure 1b, *upper*) to become a unique target which ensures accuracy of the joining reaction through the insertion of a single base pair between the abutted ends [7]. This accuracy is essential for the formation of an MCJ with a mandatory 17 bp P_junc _spacer between the -10 Pribnow box and an outwardly reading -35 motif in the right end [19]. Despite these changes in the catalytic domain of IRL which suppress donor function in Step I, IRL does possess the donor function [19] needed for strand transfer to the target site in the Step II SC.

IS*3 *family TPases have been identified as members of the TPase/retroviral integrase superfamily (referred to as RISF) of polynucleotidyl transferases [9,24-27] and functional comparisons of their protein-DNA interactions with those of other RISF TPases should be useful. To date, a complete and comparative biophysical analysis of the protein-DNA interactions in fully formed Step I and Step II SCs with protein complexed to the protein binding and catalytic domains of the inverted repeats (IRs) has not been reported for any IS*3 *family member or other circle-forming elements, primarily due to the difficulty in isolating full length proteins capable of binding efficiently and generating fully formed complexes with the IRs [8,28]. Partial footprints of the ends have however been carried out with cell-free extracts in IS*2 *[5] and similar analyses carried out with the N-terminal half of the truncated protein have been reported for IS*911 *[8,15,17] and IS30 [29]. In order to carry out a detailed biophysical study with fully formed complexes in IS*2 *it was first necessary to resolve the problem of the intractable insolubility of the TPase.

We report here a protocol utilizing a green fluorescent protein (GFPuv) tag that generates an IS*2 *TPase derivative that functions normally *in vivo*. We show for the first time that preparation under native conditions results in the recovery of a full length, soluble derivative that, when partially purified, binds very efficiently to cognate DNA sequences *in vitro*. This binding utilizes residues at both the N- and C-termini of the protein and is shown elsewhere to generate fully formed SCs with double stranded cognate IRR, IRL and MCJ sequences, with TPase bound to both the protein binding and catalytic domains of the ends (Lewis *et al*, Protein-DNA interactions define the mechanistic aspects of circle formation and insertion reactions in IS*2 *transposition, submitted).

Although aspects of structure-function relationships of the IS*2 *and IS*911 *TPases have been reported [30-34], we show here, using the GFP-tagged TPase derivative, that mutations which confer gain- or loss-of-function that are readily recovered in all of the principal domains of the protein (for examples, see Table 1) have been used to confirm, extend and further refine these structure-function relationships in IS*2 *and other IS*3 *family TPases. In addition, we have been able to describe the role of a residue whose mutation appears to have consequences primarily beyond its domain. Specifically, first an N-terminal 3-helix (H + HTH) bundle constitutes a binding domain whose architecture includes the HTH motif in helices 2 and 3 and possesses at least one residue in helix 3 which appears to play a more global role by affecting cleavage reactions in the catalytic active site (CAS). Adjacent to this, is an atypical leucine zipper-like motif, null mutations of which have allowed us to decipher its mode of function in oligomerization and binding. Within the C-terminal half of the protein, a middle domain is located adjacent to a 5α helix/5β strand secondary structure motif, the CAS, which is highly conserved in the RISF. Gain- and loss-of-function mutations in this latter domain help describe its role in regional binding (that is, to the catalytic domain of the ends (Lewis *et al*, Protein-DNA interactions define the mechanistic aspects of circle formation and insertion reactions in IS*2 *transposition, submitted) and global binding of the protein; but equally importantly, they give credence to the supposition that, at the tertiary level, the organization and function of the CAS is similar to that of the three dimensional α/β/α catalytic core motif of proteins of the RISF.

**Table 1:** Distribution, from 23 recovered mutants, of 25 randomly induced mutations in the four domains of the IS*2 *OrfAB TPase

					Domains^a^					
**Wild type/** **Mutants^b^**	**1-14**	**13-60** **H+HTH**	**61-69**	**70-103** **LZ-L**	**104-206** **MI**	**207** **-235**	**236-292** **(D240)** **CAS**	**293-334** **(D306)** **CAS**	**335-398** **(E342)** **CAS**	**399-409**

Wild type										
03								R291H		
04		A42T								
06				Q79L						
07				N94D						
09		R50H								
13		S57G								
18		A42T		L97H						
22									A341P	
24							L266P			
28		S44N								
29		L58I								
31								V301M		
34		R13H								
36		R37Q/S44N								
37		W49R								
38									A341T	
40		V35L								
68							H267D			
71									E391K	
94				K89M						
96							W237R			
101					V179L					
106				L83V						

^a^Domains of the IS*2*OrfAB TPase, deduced from these studies are as follows: H + HTH: the Binding domain; LZ-L: Leucine zipper-like oligomerization motif; MI: the middle interval; CAS: putative catalytic active site which extends from residue 236-398. The locations of the three catalytic carboxylates are shown. The sequence associated with D240 includes the β strands 1-3, that with D306, β strands 4-5 and α helices 1-3 and that with E342 α helices 4-6. Intervals represented by the numbers 1-14, 61-69, 207-235, and 399-409 are most likely disordered sequences (see Figure 9a). ^b^The wild type fusion protein (OrfAB-GFP) was overexpressed from the plasmid pLLIS*2orfAB::GFP *(pLL2522). Mutant proteins were overexpressed from the GMF strains carrying similar plasmids (pLL2524-XXX i.e., from 001-110) carrying a mutagenized *orfAB *gene. Isolated GMF strains were numbered from 1-110.

## Results

### Purification of the IS*2 *TPase by conventional methods

Conventional methods for purifying active full length IS*2 *TPase under native conditions generated insoluble protein as inclusion bodies. Although standard solubilization protocols [35-37] and attempts at directed evolution [38] were unsuccessful, the protein was easily purified to homogeneity using denaturing protocols and refolded either on-column [39,40] or in solution [41-43] in native buffers. In all cases, these TPase preparations bound very poorly to oligonucleotide substrates containing the cognate IRR DNA sequence in gel-retardation studies (for example see Figure 5a, lane 2).

### Creation of an IS*2orfAB::GFP *fusion construct

**Figure 2:**
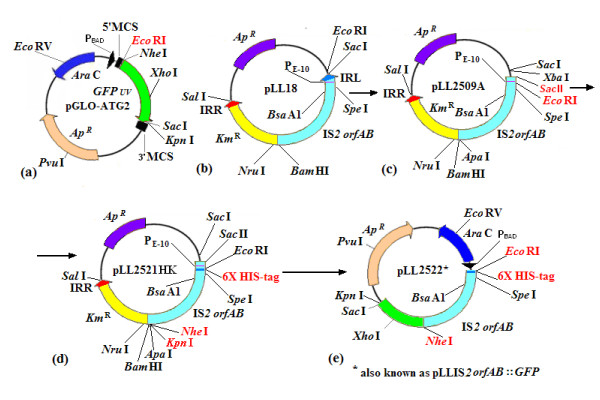
**Structure of plasmids used to create the IS*2OrfAB::GFP *fusion construct**. Modifications and alternations are indicated in red. **(a) **pGLO-ATG2, a derivative of the commercially available pGLO plasmid (Biotechnology Explorer GFP Chromatography kit, Bio-Rad Inc., Hercules, CA, USA) containing the *GFPuv *gene under the control of the P_BAD _promoter. An *Eco*RI-*Nhe*I cassetting site was created in the 5' multiple cloning site (MCS), to facilitate the cloning of the IS*2orfAB *fused frame gene. A unique *Eco*RI site was deleted from its position adjacent to the GFP stop codon and transferred to a position downstream of the P_BAD _promoter and 9 bp from an existing *Nhe*I site which encodes the first two amino acids of GFP. The mutagenizing primer for this last step also deleted the GFP start codon to create pGLO-ATG2. **(b) **pLL18, a pUC19 derivative with IS*2 *carrying the Km^r ^reporter gene [6]. IS*2 *in this construct contains the engineered *orfAB *gene described in Figure 1a (*ii*). **(c) **pLL2509A was created by removing the left inverted repeats and repositioning the existing *Eco*RI site to a location downstream of the P_IRL _promoter, effectively excluding this IS*2 *endogenous promoter from subsequent cloning of the cassetted *orfAB *gene. **(d) **pLL2521HK was created by the successive steps of adding (i) the 3'-located cassetting *Nhe*I site which included the removal of the *orfAB *stop codon and (ii) the 6XHIS-Tag, downstream of the *Eco*RI cassetting site. **(e) **pLL2522 was formed when the *Nhe*I-*Eco*RI cassetted *orfAB *(part d) was cloned into the corresponding 5' cloning site of pGLO-ATG2 (part a). bp: basepair; GFP: green fluorescent protein; IS: insertion sequences.

**Figure 3:**
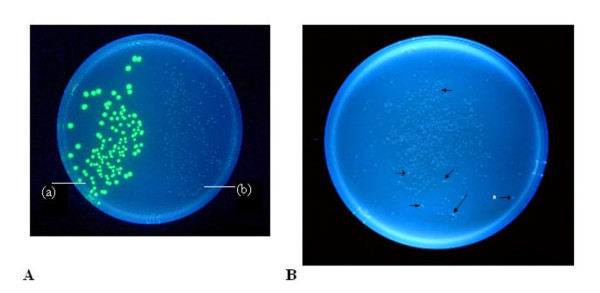
**Comparative growth and fluorescence of colonies with the pGLO, pLL2522 and pLL 2524-XXX plasmids**. **(A) **Contrasting growth patterns of colonies of XL1 Blue cells of *E. coli *(Stratagene Inc.) transformed with (a) the pGLO plasmid and (b) the pLL2522 (IS*2orfAB*::*GFP*) plasmid. Cells were plated on lysogeny broth (LB) plus carbenicillin and arabinose, incubated at 37°C for 48 hours and irradiated with UV light. **(B) **XL1 Blue cells transformed with the ligation products generated by cloning PCR products recovered from the Genemorph II Random mutagenesis of IS*2orfAB *DNA, into the *Eco*RI/*Nhe*I sites of pGLO-ATG2. Colonies were generated as described above and viewed after 72 hours at 37°C. Arrows identify the faster growing more brightly fluorescing colonies, the vast majority of which contained plasmids pLL2524-XXX (IS*2orfAB*::*GFP*-GMF) with loss-of-function mutations in the *orfAB *gene. Isolated colonies at the periphery of the Petri dish (see white asterisk) occasionally produced false positives without mutations or with silent mutations, for example, A42T. PCR: polymerase chain reaction.

Fusion of the *GFPuv *gene to the carboxy- but not the N-terminus of IS*2orfAB *generated a soluble fusion product under native conditions (see Methods). In brief, IS*2orfAB *was cloned into pGLO-ATG2 (Figure 2a), a modified version of the commercially available pGLO plasmid. The strategy was to clone an *Eco*RI-*Nhe*I cassetted version of IS*2orfAB *(Figure 2d) into the cloning sites created at the 5' end the of the *GFP *gene to generate pLL2522 (IS*2orfAB*::*GFP *clones; Figure 2e). The resulting slow growing colonies fluoresced much less intensely than control colonies carrying only the pGLO plasmid (Figure 3a).

### Overexpression of the putative IS2OrfAB-GFP fusion protein

**Figure 4:**
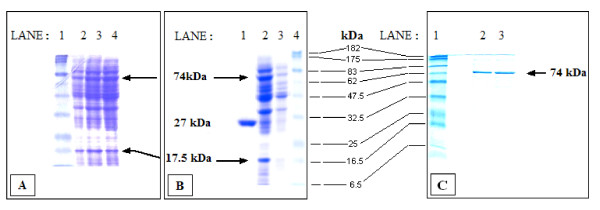
**12% SDS-PAGE analysis of proteins prepared under native conditions**. **(A) **Analysis of fluorometrically determined peak fractions from Ni-NTA gravity flow affinity chromatography purification of the 6xHis-tagged OrfAB-GFP. Lanes: 1. Prestained Protein Molecular Weight markers (New England Biolabs). 2-4. Partial purification of the 74 kDa His-tagged OrfAB-GFP fusion protein (upper arrow) from cells with the pLL2522 plasmid. The lower arrow identifies the 17.5 kDa OrfA protein generated by programmed -1 translational frameshifting. These lanes represent peak fractions (determined fluorometrically) which were run out prior to pooling. **(B) **Analysis of the pooled fractions in part (A) following concentration and dialysis (see Methods). Lanes: 1. Hydrophobic interaction chromatography purification of the 27 kDa GFP from cells with the pGLO plasmid. 2. Pooled fractions from the purification protocol. 3. Protein preparation from the pGLO-ATG2 control plasmid. 4. Prestained protein molecular weight markers. **(C)**. Purification of the 74 kDa OrfAB-GFP fusion protein to near homogeneity with the IMPACT system (New England Biolabs) from overexpression of the fused *orfAB::GFP *genes cloned into the pTWIN2 vector. The eluted protein was subjected to a polishing step on an ion exchange Hi Trap Q sepharose column (GE Healthcare Biosciences). GFP: green fluorescent protein; kDA: kiloDaltons; orf: open reading frame.

We assumed that the presence of fluorescence in colonies with the pLL2522 plasmid was an indication of a soluble fusion protein, and the supposition that the diminished fluorescence (see below) was not due to partial solubility of the protein [44] was confirmed by the presence of bright fluorescence of the supernatant after a standard native lysis procedure. Partial purification (see Methods) generated two prominent bands present in these isolates following SDS-PAGE analysis (arrows; Figure 4a, lanes 1-3 and 4b, lane 2) but absent from the control pGLO (Figure 4b, lane 1) or the pGLO-ATG2 plasmids (Figure 4b, lane 3). These were determined to be the 74 kDa fusion protein (the 46-kDa IS*2*OrfAB TPase and the 27 kDa GFP) and the 17.5 kDa OrfA protein, the product of ribosomal frameshifting [3,4]. The 74 kDa protein was also expressed from plasmid pTW2*orfAB::GFP*, where *orfAB::GFP *was cloned into a pTWIN2 vector (IMPACT; New England Biolabs, Ipswich, MA). In this case it was easily purified to near homogeneity using the manufacturer's protocol, followed by an ion exchange Q-sepharose polishing step (HiTrap Q XL, GE Healthcare, Piscataway, NJ; Figure 4c).

**Figure 5:**
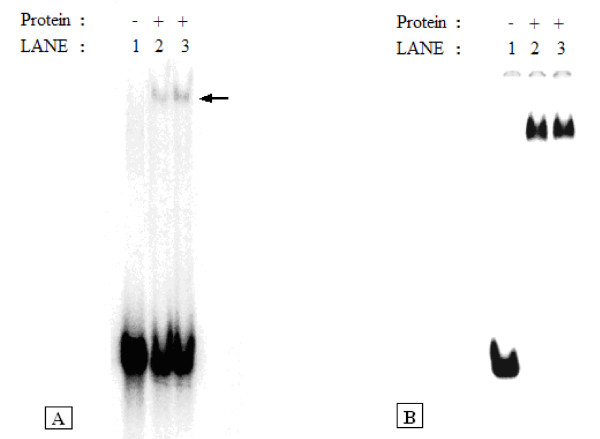
**Electrophoretic mobility shift assays using purified and partially purified preparations of the IS*2*OrfAB-GFP fusion protein**. **(A) **Purified OrfAB-GFP fusion protein preparations shown in Figure 4c and the purified native protein from refolding experiments were used in gel retardation reactions. 0.46 μM of the fusion protein and 6.02 μg of the refolded protein were reacted for 30 minutes at room temperature (20°C) with 2 nM of ^32^P-labeled annealed 87-mer oligonucleotides containing the 41 bp inverted right repeat sequence. The reactions were run at 4°C at 120 mA for 2400 Vhr in a 5% native polyacrylamide gel. The arrow shows complexes formed with low efficiency. Lanes: 1. Protein-free control. 2. Refolded native OrfAB. 3. OrfAB-GFP. **(B) **Partially purified preparations of the OrfAB-GFP fusion protein shown in Figure 4a and crude extracts from overexpression of the pTW2 OrfAB-GFP construct used in binding reactions. Approximately 80 nM of the protein from the partially purified preparations shown in Figure 4a and from the crude extracts were reacted with 2 nM of the ^32^P-labeled annealed 87-mer oligonucleotides as described in part A. The reactions were run for 1400 Vhrs at 4°C. Lanes: 1. Protein-free control. 2. Partially purified preparation of OrfAB-GFP. 3. Crude extract from the overexpressed pTW2 OrfAB-GFP plasmid. Bp: base pairs; GFP: green fluorescent protein; orf: open reading frame; Vhr: volt hour.

### Electrophoretic mobility shift assays with IS*2*OrfAB-GFP

Preparations of the OrfAB-GFP fusion protein purified to near homogeneity also bound poorly to cognate DNA sequences in gel retardation assays (Figure 5a, lane 3). Neither OrfA nor host factors, such as the bacterial histone-like protein, HU and integration host factor [45-47] enhanced binding efficiency (data not shown). On the other hand, the partially purified preparations of OrfAB-GFP shown in Figure 4a, lanes 2-4, generated results in which all of the DNA was driven into the complex (Figure 5b, lane 2). A similar result was obtained with the crude extract from the overexpression of pTW2*orfAB::GFP *(Figure 5b, lane 3). The multimeric nature of these complexes has been demonstrated in concurrent footprinting studies in which complexes similar to those shown in Figure 5b were created with MCJ DNA substrates containing abutted IRR and IRL ends. There, the protein binding domains and the catalytic domains of the two ends were protected along their entire lengths, suggesting that the complex consisted of at least a dimer (Lewis *et al*, Protein-DNA interactions define the mechanistic aspects of circle formation and insertion reactions in IS2 transposition, submitted).

### Fluorescence levels can be used to isolate IS*2 *TPase loss-of-function mutants leading to a structure-function analysis of the protein

We asked whether loss-of-function mutants of the IS*2 *TPase could be isolated as faster growing more brightly fluorescing colonies in order to test the idea that the low level of fluorescence of slow growing colonies with the pLL2522 plasmid might be due to the toxicity of the fusion protein, as well as to explore the possibility that we could obtain and analyze random mutations along the entire length of the protein. Random mutagenesis of IS2*orfAB *was accomplished with the PCR-based Genemorph II Random Mutagenesis kit (Stratagene, Santa Clara, CA) using very low, low and medium mutation rates. PCR products were cloned into the *Eco*RI/*Nhe*I sites of pGLO-ATG2 and the ligation products transformed into XL1blue cells (Stratagene). After 72 hours at 37°C, faster growing, more brightly fluorescing colonies were observed among a background of less intensely fluorescing colonies (Figure 3b). Recovery and analysis of the plasmids pLL2524-XXX (that is, 001-110) from these brighter fluorescing isolates (referred to here as GMF strains 1-110) showed that they carried mutations at frequencies which corresponded to the protocol-based mutation rates.

**Figure 6:**
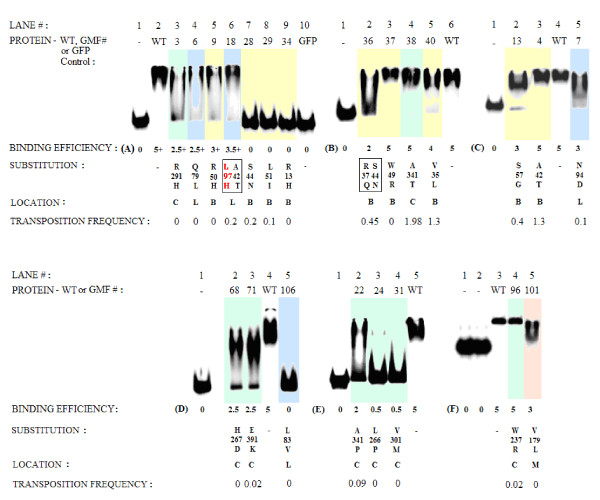
**Electrophoretic mobility shift assays**. Binding efficiencies of the IS*2*OrfAB Transposase derivatives from 22 randomly induced mutants. Reactions were carried out for 30 minutes at 20°C, with 10 nM of ^32^P-labeled annealed 50-mer oligonucleotides (except where stated in part (f) below) containing the inverted right repeat sequence and 0.11 μM of the partially purified mutant or wild type IS*2*OrfAB-GFP protein derivatives (see Methods). Domain locations of the substitutions are color-coded and identified by a single letter code, that is, the binding domain (B) yellow, the leucine zipper-like (L) blue, the catalytic active site (C) green, and the middle interval (M) orange. Reactions were separated on 5% native polyacrylamide gels at 4°C at 120 mA as follows: (a) 450 Vhrs. (b) 420 Vhrs (c) 300 Vhrs (d) 450 Vhrs (e) 450 Vhrs (f) 12% native PAGE for 300 Vhrs using 87-mer annealed oligonucleotides. Binding efficiencies are identified as follows: 5 = Identical to that of the wild type, that is, absence of any dissociation of the complex. 4.5 = a slight loss of compactness of the undissociated complex seen in the wild type control. 4.0 = as in 4.5 but with a faster migrating tail of dissociated complexes. 3.5 = as in 4.0 but with a more prominent faster migrating tail of dissociated complexes. 3.0 = significant loss of compactness of the complex with a small amount of uncomplexed DNA. 2.5 = as in 3.0 but with significantly more uncomplexed DNA. 2.0 = as in 3.0 but mostly composed of uncomplexed DNA. 0.5 = mostly composed of uncomplexed DNA with a small tail of dissociated complex. 0 = no complex formation, identical to that of the protein-free controls (lane 1 in each panel) or the GFP control (part a lane 10). Double mutations are indicated within rectangular boxes. For GMF 18 the operative mutation, L97H, is shown in red (gel c, lane 4). GFP: green fluorescent protein; orf: open reading frame; Vhr: volt hour

From the 110 brightly fluorescing colonies which were isolated, twenty one *orfAB *sequences containing single mutations and two with interesting double mutations were successfully analyzed for the nature of their amino acid substitutions (Table 1) and for the corresponding effect of the substitutions on transposition frequencies (Table 2) as determined by a *lacZ *papillation assay [48]. In addition, the relative binding efficiencies of the TPase to the cognate IRR DNA sequence from 22 of the 23 mutants were determined on electrophoretic mobility shift assay (EMSA) gels (Figure 6 and Tables 1 and 2).

**Table 2:** *In vitro *electrophoretic mobility shift assays, binding efficiencies and *in vivo LacZ *papillation assay-determined transposition frequencies from IS*2 *wild type and mutant (GMF^a, d^) isolates

Column number	1	2	3	4	5	6	7	8	9
Row number	**Description of wild type and mutant GMF plasmids/strains**.	Binding efficiency^a^	Description or mutation	Domain-location of mutations^b^	Total number of colonies	Number of trials (n)	Number of colonies with papillae	Total number of papillae	Transposition frequency^c^
1	pUH2523ΔorfAB^c^	-	Null	-	83	5	14	15	0.18 ± 0.16
2	pUH2509^d^	-	WT OrfAB	-	96	2	66	137	1.25 ± 0.23
3	pLL2522^a^/pUH2523^d^	5.0	WT fusion protein	-	174	6	95	254	1.28 ± 0.09
4	pLL2524^a^/pUH2524-004^d^	5.0	A42T	H + HTH	43	3	24	64	1.31 ± 0.20
5	-009	3.0	R50H	H + HTH	18	1	2	2	0.00
6	-013	3.0	S57G	H + HTH	66	4	26	33	0.32 ± 0.17
7	-028	0.0	S44N	H + HTH	59	2	18	24	0.23 ± 0.08
8	-029	0.0	L58I	H + HTH	68	2	14	17	0.07 ± 0.07
9	-034	0.0	R13H	H + HTH	97	2	12	12	0.00
10	-036	2.0	R37Q/S44N	H + HTH	162	4	51	73	0.27 ± 0.11
11	-037	5.0	W49R	H + HTH	62	2	4	4	0.00
12	-040	4.0	V35L	H + HTH	52	3	38	75	1.26 ± 0.18
13	-006	2.5	Q79L	LZ-L	137	3	10	10	0.00
14	-007	3.0	N94D	LZ-L	52	2	14	16	0.13 ± 0.09
15	-018	3.5	A42T/L97H^e^	LZ-L	44	2	13	17	0.21 ± 0.15
16	-094	ND	K89M	LZ-L	43	3	2	2	0.00
17	-106	0.0	L83V	LZ-L	133	4	4	4	0.00
18	-003	2.5	R291H	CAS	59	1	11	10	0.00
19	-022	2.0	A341P	CAS	88	5	16	18	0.09 ± 0.11
20	-024	0.5	L266P	CAS	34	2	4	4	0.00
21	-031	0.5	V301M	CAS	60	2	2	2	0.00
22	-038	5.0	A341T	CAS	37	2	30	80	1.98 ± 0.21
23	-068	2.5	H267D	CAS	106	3	17	19	0.00
24	-071	2.5	E391K	CAS	139	4	19	28	0.20 ± 0.26
25	-096	5.0	W237R	CAS	80	4	10	16	0.02 ± 0.05
26	-101	3.0	V179L	MI	113	4	7	10	0.00

^a^Binding efficiencies were determined from electrophoretic mobility shift assays as described in the Methods section and illustrated in Figure 6. The wild type OrfAB-GFP fusion protein was expressed from pLL2522 and mutant OrfAB-GFP-GMF proteins from pLL2524-XXX plasmids (1-110). ^b^Domains and locations of mutations are as described in Table 1. ^c^Transposition frequencies were calculated as the number of papillae per colony (column 8 divided by column 5) minus the background frequency of transposition calculated from the null mutation (row 1). Frequencies shown for the mutants reflect only their contributions to the observed results (column 8/column 5). When 0.0 is listed, the observed result is less than the background frequency probably due to experimental error or variation in the count which may have been a function of sample size. The null mutation was derived from self ligation following an *Mfe*I digestion which removed most of the IS*2orfAB::GFP *fusion from the pUH2523 plasmid. The background frequency of transposition from the null mutant is likely due to the presence of IS*2 *copies on the chromosome of JM105 into which plasmids used in the *LacZ *assay were transformed (see Methods). ^d^Plasmids used to determine transposition frequencies by means of the *LacZ *papillation assay were pUH2509 for the WT OrfAB protein, pUH2523 for the WT OrfAB-GFP protein and pUH2524-XXX (1-110) for the mutant OrfAB-GFP-GMF proteins. ^e^The phenotype of GMF 18 is attributed to the L97H substitution since both the binding efficiency and the transposition frequency of the A42T substitution (row 4) do not differ statistically from those of the wild type (row 3). CAS: putative catalytic active site; H + HTH: the binding domain; LZ-L: leucine zipper-like oligomerization motif; MI: the middle interval; WT: wild type.

### Sequence analysis of the wild type IS*2 *TPase and secondary structure analysis of the IS*3 *family TPases

**Figure 7:**
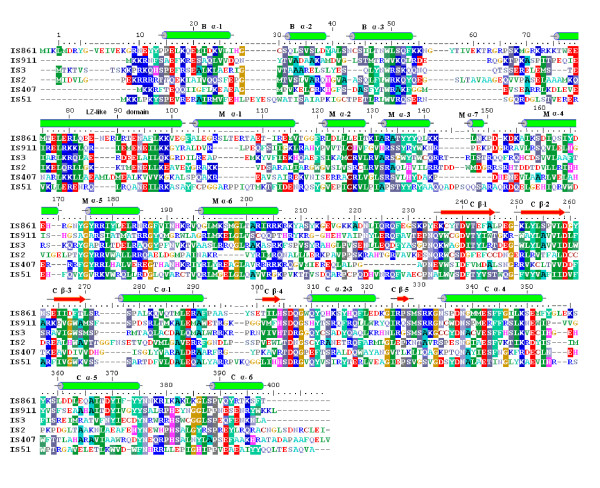
**Alignment of OrfAB sequences from IS*3*-family sub-groups correlated with secondary structure data of IS*2***. Sequences in descending order, IS*861 *(IS*150 *subgroup), IS*3*, IS*911 *(IS*3 *subgroup), IS*2*, IS*407 *and IS*51 *were aligned using the ClustalW2 multiple sequence alignment tool [50]. Coordinates above the sequences are those of IS*2*. Amino acid groups are color coded as follows: Red - acidic residues; blue - basic residues; green - non-polar hydrophobics; cyan - aromatics (Y and F); dark green - tryptophan; gray - proline; light purple - amides; blue-gray - small polar; aquamarine - small non-polar; ochre - glycine; magenta - histidine and brown - cysteine. Secondary structure elements (green cylinders for α helices and red arrows for β strands) for IS*2 *were determined by the Protein Structure Prediction Protocol (see Figure 8) and are shown above the sequences for the N-terminus of the protein as B α1-3 (putative binding domain), the putative leucine zipper-like domain and the middle interval (elements M α1-7). In the C-terminal half of the sequences, elements of a putative catalytic active site motif are identified as C β 1-5 and C α 1-6. IS: insertion sequence.

**Figure 8:**
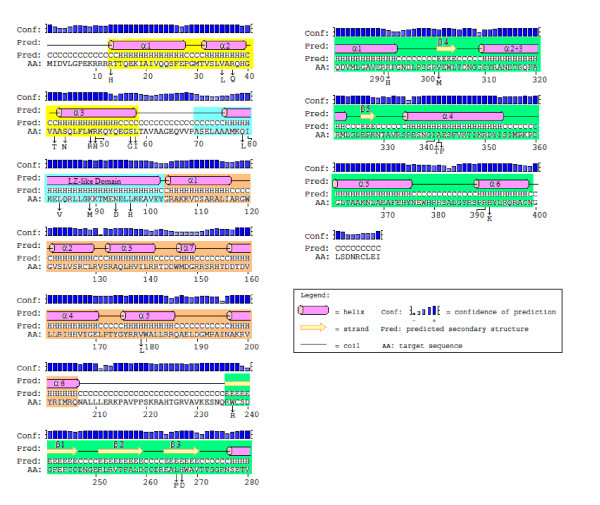
**Secondary structure elements of the IS*2 *OrfAB TPase**. Elements were generated by the Protein Structure Prediction server [51]. The transposase (TPase) sequence has been color coded to identify the four putative domains; binding (yellow), oligomerization (leucine zipper-like; blue), a middle interval (orange) and the catalytic active site (CAS; green). The numbering of α helix #7 in the middle interval is designed here to reflect the alignment of the six principal α helices found in the IS*3 *family (Figure 10a). Numbering of α helices 2 and 3 in the CAS reflects the organization of the aligned elements in TPase and integrase sequences of the TPase/retroviral integrase superfamily (Figure 9c). Vertical arrows and substituted amino acids identify the locations of the 23 substitutions within the secondary structures of the IS*2 *TPase. CAS: catalytic active site; TPase: transposase.

The wild type IS*2orfAB *DNA sequence and those of five other members (IS*861*, IS*3*, IS*911*, IS*407*, and IS*51*) of the five principal sub-groups of the IS*3 *family [1,30] were translated into the protein sequences using the ExPASy SWISS PROT translation toolkit [49]. These sequences were aligned using the ClustalW2 multiple sequence alignment tool [50] producing many groups of short aligned sequences (Figure 7) which were then analyzed for their secondary structure (Figure 8) using the Protein Structure Prediction (PSIPRED) Server [51]. Figure 7 merges the sequence alignment data and the secondary structure data for IS*2 *and describes a pattern that is essentially conserved in all of the five principal subgroups of the IS*3 *family (data not shown).

**Figure 9:**
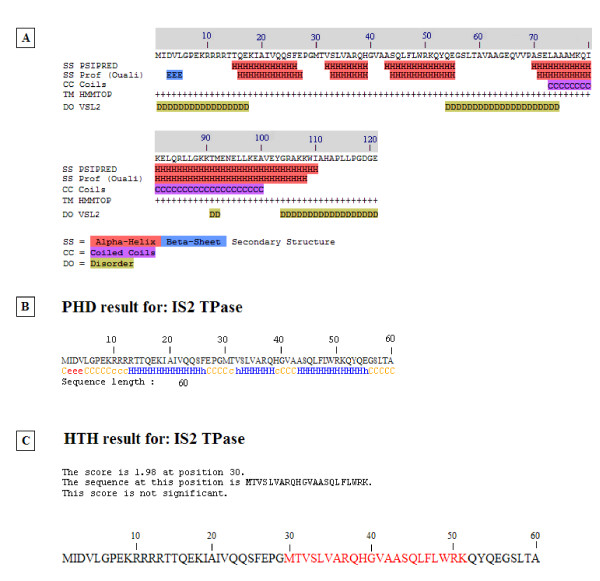
**Secondary structure predictions for the first 120 amino acids of the IS*2 *OrfAB TPase**. **(A) **Comparison of secondary structure predictions based on the Protein Structure Prediction server protocol [51] and the PROF Secondary Structure Protocol [53]. The PCOILS analysis for coiled coils [57,58] is also shown. Disordered regions (D) determined by the VSL2 predictor package from the DisProt database [111,112] correspond well with these secondary structure predictions. **(B) **Secondary structure analysis of the first 60 amino acids of the IS*2 *TPase generated by the Pole Bioinformatique Lyonnais [54] PHD Secondary Structure Analysis algorithm [55]. H/h = alpha helix; C/c = random coil and e = extended strand. **(C) **Identification of a putative HTH motif in the first 60 amino acids of the IS*2 *TPase generated by the Pole Bioinformatique Lyonnais HTH Determination Algorithm of Dodd and Egan [56]. TPase: transposase.

Although DNA binding domains in TPases have long been identified at their N-termini [52] and an HTH motif for the IS*911 *TPase in the IS*3 *family has been confirmed experimentally by Rousseau *et al. *[34], the precise nature at the secondary structure level of all elements which contribute to the three-dimensional architecture of the binding domain in this family, and specifically in IS*2*, has not been demonstrated (see [5,33]). We asked whether the three N-terminal α helices might comprise such a binding domain in IS*2 *and used the PSIPRED server [53] (Figure 9a) and the PHD secondary structure analysis algorithm (Pole Bioinformatique Lyonnais (PBIL; [54,55]) to arrive at a consensus that the location of three α helices in a putative binding domain in the IS*2 *TPase was somewhere between residues 13 and 55 (Figure 9b). In addition, a PBIL-HTH Determination Algorithm based on the protocol of Dodd and Egan [56] detected an HTH motif at residues 30-51 (Figure 9c) corresponding approximately to helices 2 and 3 in Figures 8, 9a and 9b. Similar predictions have been made for the existence of an HTH motif in IS*2 *(residues 31-50) [5,33] and in the IS*3 *family (including IS*2*, residues 30-55) [34], with the assumption in the latter study that a third N-terminal helix might form part of the binding domain. In this study we show through randomly recovered mutations that the binding domain of the IS*2 *TPase at a secondary level consists of a three-helix H + HTH bundle and provide evidence for the precise locations of the three helices.

A PCOILS analysis for coiled coils [57,58] predicted the presence of a coiled coil motif (Figure 9a) in the IS*2 *TPase between residues 73 and 100. Lei and Hu [33], using deletion derivatives of IS*2 *OrfA, showed that a sequence between residues 58 and 105 was responsible for dimerization and they as well as Haren *et al. *[30], predicted that the sequence between residues 73 and 100 of IS*2 *OrfA possessed an atypical heptad repeat showing some similarities to the canonical leucine zipper (LZ) of DNA binding proteins. In this study, however, a probe for the potential for a LZ within the first 120 residues of IS*2 *OrfAB was scored at a probability of zero using the 2ZIP server [59] even though the existence of a coiled coil domain between residues 73 and 100 was confirmed with a probability of 0.8 to 1.0. Here, we show through the use of loss-of-function point mutations how this sequence functions as an LZ-like motif and describe its role in the oligomerization, DNA binding and transposition properties of the IS*2 *TPase.

The alignment corresponding to IS*2 *residues 103 to 400 in Figure 7 matches that previously published for the IS*3 *family TPases and the retroviral integrases [60], as well as for the IS*3*, IS*4 *and IS*6*-family TPases and integrases from several retroelements residues 236-354 [61]. The latter sequence, the CAS, is characterized by the presence of an invariant triad of catalytic carboxylases, the D, D(35)E motif [9,27,62,63]. We asked what degree of correlation might exist between the aligned residues 101 to 400 in Figure 7 and a structure-based alignment of the sequences of the α helices and β strands generated by PSIPRED analysis in Figure 8; that is, how similar would these elements be in sequence and length in the IS*3 *family TPases and in the HIV-1 and Rous sarcoma virus (RSV) integrases.

**Figure 10:**
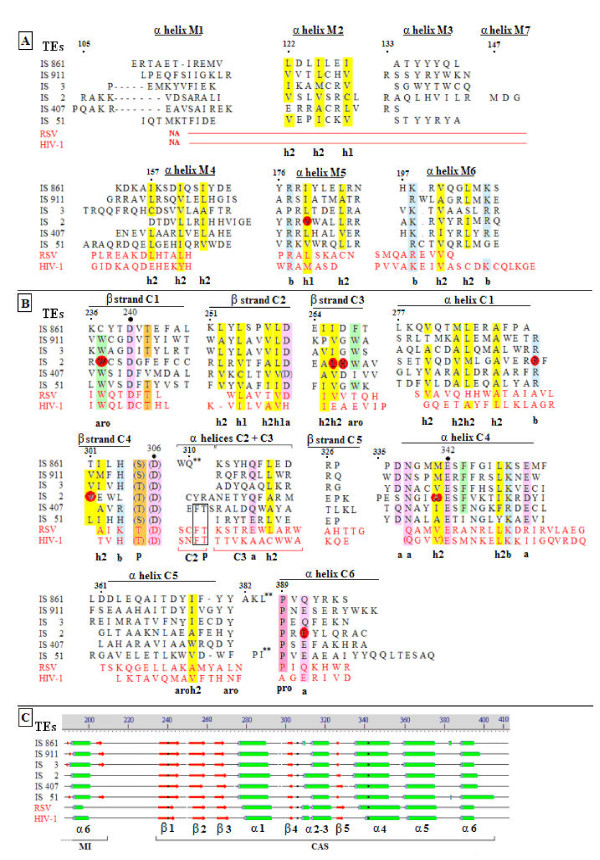
**Structure-based alignments of middle interval and catalytic active site elements of IS*3*-family transposases and HIV-1 and Rous sarcoma virus integrases**. **(A) **α helices identified in the middle interval of the IS*2 *transposase (TPase) and the corresponding sequences of five other members of the principal sub-groups in the IS*3 *family. Where applicable, the sequences of corresponding elements in the Rous sarcoma virus (RSV) and the HIV-1 were also aligned (red lettering). All coordinates are those of IS*2*. Functionally conserved non-polar hydrophobic residues are highlighted in yellow and identified as h1 and h2 (Methods - alignment tools). Functionally conserved basic residues (b) are highlighted in blue. NA = no alignments identified in the integrases of RSV and HIV-1. **(B) **α helices and β strands in the catalytic active sites (CASs) of the TPases of IS*2*, five other IS*3 *family members, and the integrases of RSV and HIV-1 (red lettering). Functionally conserved hydrophobic and basic residues are identified as described in part A. In addition, functionally conserved acidic residues or their amides (a) are highlighted in purple, non-polar aromatics (aro) in green, polar serines and/or threonines (p) in orange and prolines (pro) in mauve. DDE residues are indicated by large black dots. Sequences in parentheses are not components of the α helices or β strands. α helix 2 (2+3) in the TPases aligns with helices 2 and 3 in the integrases. Residues conserved in α helix 2 of the integrases and in its remnants in IS*407*, are enclosed in a black rectangle. Large double asterisks indicate short α helices with no homology to other sequences (see part C graphic). Substitutions are indicated by red ovals; twin ovals indicate A341P and A341T. **(C) **Graphic alignment of α helices and β strands of the CASs of the TPases of IS*2 *and five other members of the IS*3 *family and of the integrases of RSV and HIV-1. Black dots within the elements represent the positions of the DDE triad. DDE: the catalytic triad of two aspartates and a glutamate; CAS: catalytic active site; IS: insertion sequence; RSV: Rous sarcoma virus; TPase: transposase.

Of the six alpha helices in a middle interval (residues 105 to 210 of IS*2*), from all six TPases in the IS*3 *family sub-groups (Figure 10a), only α helices 2, 5 and 6 were well aligned. Only α helices 4, 5 and 6 in the IS*3 *family, located just upstream of the CAS (Figure 8), aligned with the NH_2_-terminal α helices of the integrases.

Structure-based sequence alignments of residues corresponding to residues 236 to 398 in IS*2 *for IS*3 *family TPases and the HIV-1 and RSV integrases showed a series of five well-aligned α helices and five equally well-aligned β strands (Figure 10b), showing almost perfect conservation in their lengths, with high levels of identity (the presence of the same amino acid in at least 85% of the eight sequences) and high proportions of functionally conserved residues per element (approximately 50% in the β strands and 25% in the α helices). The significance of this in this study is that all but one of the eight random mutations recovered in this domain occurred at these conserved residues.

These α helices and β strands occur in a conserved order (Figure 10c) characteristic of the integrases and of the TPases with the DDE motif of two aspartates and a glutamate, for example, Mu [64], Tn*5 *and the IS*1 *family [65,66]. In IS*3 *family TPases, α helices 2 and 3 in the integrases are present as a single helix (α helix 2) and it is interesting that remnants of α helix 2 of the integrases are seen in IS*2 *and IS*407 *but specifically in IS*407*, as two well-conserved residues in the first three amino acids of the single α helix (Figure 10b). In IS*911 *of the IS*3 *family, this group of tightly conserved elements has been proposed to be the putative CAS [2,24,34].

The three-dimensional structure of this unit, the catalytic core, has been demonstrated in several members of the TPase/RISF, including the TPases of the DDE family, such as Mu [64] and Tn5 [67], the integrases, such as HIV-1 [68-71] and the avian (ASV) and Rous (RSV) Sarcoma viruses [72,73] and other nucleases, for example, RNase H1 [74,75] and RuvC [76]. For comprehensive reviews see [25,26,77]. This catalytic core is characterized by a five-stranded partially buried β sheet of mixed parallel and antiparallel elements with a polar face, with six α helices distributed on either side of it. The two aspartate residues of the DDE catalytic triad are located on adjacent strands of the β sheet (numbers 1 and 4) with the glutamate residue assigned to the closely located α helix 4 [78]. We show here that randomly induced mutations in this putative catalytic core that affected residues other than the DDE alter the function of this motif in both positive and negative ways, identifying additional signatures characteristic of the catalytic core and supporting the intuitive contention that, in the IS*3 *family, it is organized and functions like the three-dimensional structure in the RISF; additional mutations also provide insights into its role in both the regional and the global binding strategies of the protein.

### Effect of TPase mutations on TPase binding efficiencies and on *in vivo *transposition frequencies of IS*2*

Eleven of the twenty-five mutations (from the twenty-one single mutants and two double mutants) were within the putative binding domain, five were located in the coiled coil domain, eight in the putative CAS and one in the middle interval (Table 1; see also Figure 8 for an overview of the locations of these mutations within the secondary structures of the TPase). The binding efficiencies of the partially purified TPases of 22 of the mutant proteins were studied by EMSA (Figure 6) using a pair of annealed oligomers (50 bp in length) containing 41 bp of cognate DNA of the IRR [6]. The substrate was labeled at the 5' end of the upper strand with γ^32^P (see Methods). A summary of the binding efficiencies together with results of *in vivo *transposition frequencies of all 23 mutants (determined from *lacZ *transposition assays) is shown in Table 2 .

### The putative binding domain

Nine mutants with substitutions in the putative binding domain are described in Table 2 (rows 4-12). Binding data are shown in the EMSA gel (Figure 6, yellow highlights). Proteins from three of the mutants, GMF isolates 28 (S44N), 29 (L58I) and 34 (R13H) (Figure 6a, lanes 7-9) formed no complexes, indicative of structural defects. The TPase from the double mutant, GMF 36 (R37Q/S44N- Figure 6b, lane 2), however, showed a partially restored, unstable, dissociated complex, absent in isolate 28 (S44N). Two GMF isolates, 9 (R50H; Figure 6a, lane 5) and 13 (S57G; Figure 6c, lane 2) also produced proteins which formed mostly dissociated complexes, likely indicative of deficiencies in binding reactions to the DNA substrate (see discussion). All six of the mutants with TPases completely defective or deficient in binding, (GMF isolates 9, 13, 28, 29, 34 and 36) had significantly reduced or no detectable levels of transposition (Table 2, rows 5-10). The remaining three mutants with substitutions in the putative binding domain, GMF isolates 4 (A42T; Figure 6c, lane 3), 37 (W49R) and 40 (V35L) (Figure 6b, lanes 3 and 5) showed marginal or no observable effects on binding efficiency. Two of these three mutants, GMF isolates 4 (A42T) and 40 (V35L) (Table 2, rows 4 & 12) had *in vivo *transposition frequencies (approximately 1.3) that were statistically comparable to those of the wild type controls, two versions of which, one fused to GFP (Table 2, row 3) and the other not (Table 2, row 2), showed identical transposition frequencies within experimental error.

The third mutant with little or no loss of binding efficiency, GMF 37, (W49R) was the single exception to the consistency in the relationship between binding efficiency and transposition frequency described above (Table 2, row 11). While this TPase derivative was quite proficient in binding to the substrate, the substitution completely abolished transposition. The apparent inconsistency in these properties of GMF 37 can be explained by the fact that W49 in IS*2*, which is one of the most highly conserved residues in the IS*3 *family (Figure 7 and [34]) and is also conserved in the homeodomain proteins [79], may play a more global role in effecting transposition. It may not simply be limited to a binding domain function and is not likely to be involved in DNA sequence recognition in helix 3 (see discussion).

**Figure 11:**
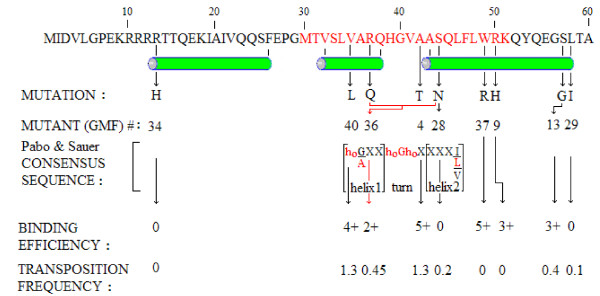
**Analysis of locations and phenotypes of nine randomly induced substitutions in the binding domain of IS*2*OrfAB**. Location of the three α helix bundle which constitutes the binding domain (green cylinders) is based on the prediction of the PBIL-PHD Secondary Structure Analysis Algorithm ([55]; see Figure 9b). The sequence in red indicates the prospective HTH motif identified by the PBIL- HTH Determination Algorithm of Dodd and Egan [56]. The Pabo and Sauer [95] consensus sequence for prokaryotic HTH motifs is shown within the large brackets and correlates well with this prospective motif (red lettering). Four of the nine mutations fell within this 12-residue consensus sequence including the double mutation represented by the combination of the red bracket and the hooked arrow. The phenotype of this double mutation is indicated by the vertical red arrow. Binding efficiencies are as described in Figure 6 and transposition frequencies were calculated as described in Table 2.

The abolition of both DNA binding and *in vivo *transposition in R13H and L58I (Table 2, rows 8 and 9) and the significant reduction in transposition frequency and binding in S57G (Table 2, row 6), suggest that the architecture of the binding domain consists of a three helix bundle encompassing residues 13 to 58. Furthermore, the ability of the R37Q/S44N double substitution in helices 2 and 3 (Table 2, row 10) to partially restore both the binding and transposition lacking in S44N, suggests that they may be involved in the H-bonded stabilization of the two helices where the HTH motif may be located (see Figure 11 and the discussion section for a complete elaboration of these ideas).

### The coiled coil motif

Five of the randomly induced mutations (in GMF isolates 6, 7, 18, 94 and 106) fell into the coiled coil segment (Table 2, rows 13-17 and Figure 10, blue highlights). Although isolate GMF 18 carries the double substitutions A42T+L97H, its phenotype, that is the loss of transposition and an unstable complex (Table 2, row 15; Figure 6a, lane 6) should be allocated to L97H, since analysis of the A42T mutation showed that the transposition frequency of the GMF 4 mutant and the binding efficiency of its protein are identical to those of the wild type. Another mutant, GMF 106 (L83V; see Figure 6d, lane 5), showed complete loss of binding proficiency and two others, GMF 6 (Q79L; Figure 6a, lane 4) and 7 (N94D; Figure 6c, lane 5) showed marked dissociation of their complexes in the EMSA gel. All five mutations effectively eliminated transposition (Table 2, rows 13-17).

**Figure 12:**
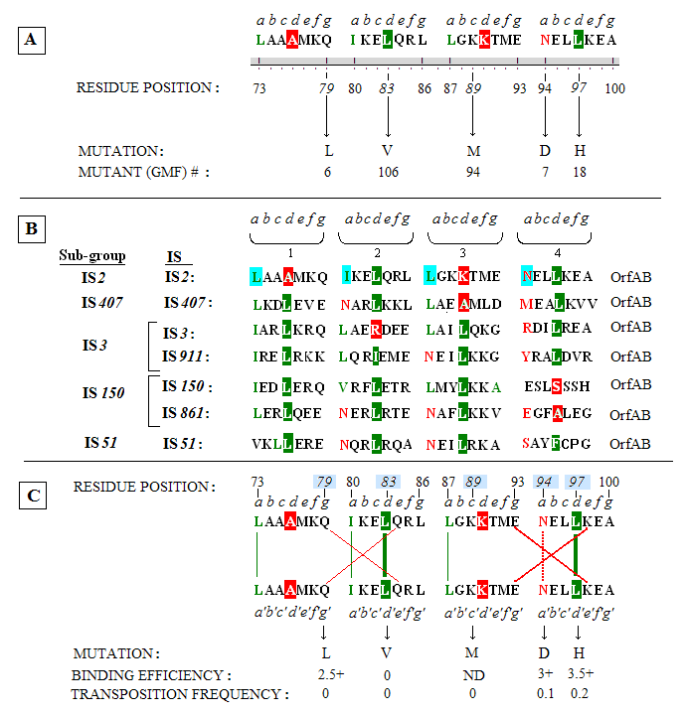
**Analysis of the coiled coil domain in IS*2*OrfAB aligned with similar domains in the IS*3 *family**. **(A) **The coiled coil sequence in IS*2 *identified by the PCOILS analysis of coiled coils [57,58] annotated to show the four putative heptad repeats of a leucine zipper-like motif. Italicized letters **a **to **g **represent the repeated positions within each heptad. The critical **d **positions which favor hydrophobic leucines are highlighted in green (or in red for a non-canonical amino acid). The **a**-located buried asparagine (N94) is shown in red while green lettering identifies the three canonical **a**-located hydrophobics. The five randomly induced mutations are indicated by arrows. The corresponding GMF mutant strain is listed beneath each mutation. **(B) **Alignment of the coiled coil domains of seven members from the five principal subgroups of the IS*3 *family showing their relationships to the putative heptads of a leucine-zipper motif. Annotation is as described in part A but for the IS*2 *sequence the **a **positions are highlighted in aqua. **(C) **Analysis of the potential of the coiled coil sequence in IS*2 *to function as a leucine zipper and the effect of mutations recovered within the motif on that function. The data suggest that the sequence which fails the 2ZIP test for a leucine zipper [59] may indeed have that function. Stabilization by the two **d**-located leucines is indicated by vertical bold green lines, by the **a**-located hydrophobics by narrow green lines and by the buried asparagine by a vertical broken red line. Weak salt bridges between glutamines in the **g **and **e **locations in heptads 1 and 2 are indicated by a large narrow-lined red × and the canonical ionic salt bridges between the **g **and **e**-located E and K residues in heptads 3 and 4, are indicated by a large bold red X. Binding efficiencies (see Figure 6) and transposition frequencies (see Table 2) are listed below the schematic. Additional annotation is as described in part A. GFP: green fluorescent protein; IS: insertion sequence.

The four heptads which make up the putative LZ motif in the IS*2 *TPase and the substitutions within them are shown in Figure 12a. This proposed LZ motif contains zipper-functional leucines in only two of the four **d **positions that are assigned to a canonical LZ [80,81]; see also the aligned sequences of predicted LZ sequences in the IS*3 *family [30]. Two of the five randomly induced substitutions in the coiled coil segment, L97H (GMF 18) and L83V (GMF 106) affected these hydrophobic residues. The three other substitutions also affected residues that are critical to the function of a LZ-like motif; Q79L (**g**) and N94D (the **a**-located buried Asn) likely affected residues that are required for inter-subunit stabilization and K89M appears to have altered a **c **position residue essential for the integrity of the helical structure. Figure 12 and the discussion section contain a detailed explanation of how all five of these randomly isolated mutations resulted in amino acid changes that would critically compromise a zipper-like function of the domain.

### The catalytic active site

Eight of the twenty-five mutations occurred in the proposed CAS of the protein (see GMF isolates 3, 22, 24, 31, 38, 68, 71 and 96 in Table 2, rows 18-25) and seven of them altered conserved residues (Figure 10b). EMSA gel reactions are shown in Figure 6 (green highlights). Three protein derivatives from GMF 22, 24 and 31 (A341P, L266P and V301M (Figure 6e, lanes 2-4) produced no complexes. Three others showed mostly dissociated complexes, GMF 3 (R291H; Figure 6a, lane 3), GMF 68 and 71 (H267D and E391K; Figure 6d, lanes 2-3). Two mutant derivatives with proficient binding reactions were GMF 38 (A341T; Figure 6b, lane 4) and GMF 96 (W237R; Figure 6f, lane 4); of these, the transposition frequency in the former was enhanced by about 50% and abolished in the latter (Table 2, rows 22 and 25). Transposition was eliminated in the six mutant derivatives with deficient or completely defective binding reactions (Table 2, rows 18-21 and 23-24). The locations of these substitutions in three α helices and three β strands of the CAS are shown in Figure 10b. Two of the eight substitutions altered residues conserved only in the IS*3 *family (R291H and V301M), one affected a non-conserved residue (H267D) and the remaining five substitutions resulted from alterations of residues conserved in the RISF.

The six TPase derivatives whose binding efficiencies were partially or completely reduced give some insight into the role of the putative catalytic core's contribution to both regional (catalytic domain) and global (catalytic and binding domains) binding of the TPase. Three mutations eliminated global binding, indicative of the structurally destabilizing effects of the substitutions. The A341P substitution located one residue from E342 of the DDE catalytic triad altered a residue at a position normally conserved for a hydrophobic amino acid in α helix 4 of the RISF. The presence of the helix-breaking proline had a devastating effect on binding and most of the DNA remained uncomplexed (Figure 6e, lane 2). Binding of the protein was completely eliminated in two other derivatives (Figure 6e, lanes 3-4). First, the L266P substitution occurred in β strand 3 where proline replaced a hydrophobic residue that is essentially conserved in the RISF; secondly, V301M changed another very hydrophobic residue that is conserved the IS*3 *family as either a valine or leucine in β strand 4 and is located adjacent to the second Asp of the DDE triad in the RISF (D306 in IS*2*).

EMSA gels of TPase derivatives with three other substitutions showed reactions in which unstable complexes were formed, suggestive of a reduction in the binding affinity of the CAS for its DNA contacts. R291H altered a positively charged residue in α helix 1, which is essentially invariant in the IS*3 *family, for one which readily assumes a neutral state (Figure 6a, lane 3). E391K substituted a basic residue for one which is essentially conserved as glutamate or glutamine in α helix 6 of the RISF. H267D substituted a negatively charged residue at a non-conserved position in β strand 3 (Figure 6d, lanes 2-3). The combined results from these six substitutions suggest that the catalytic core plays a role not only in binding to the catalytic domain of the end (unstable complexes) but that its integrity contributes to global binding proficiency of the full length protein (see Discussion).

Two mutations which did not affect binding proficiency provided insights into the role of β strand 1 and α helix 4 in facilitating the catalytic functions of the IS*2 *TPase (Table 2, rows 22 and 25). The 50% increase in transposition frequency of the mutant with the A341T mutation likely results from the substitution of a polar residue at this conserved hydrophobic position in the RISF, creating the potential for an additional specific or stochastic contact with the terminus possessing the CA-3' dinucleotide. The W237R mutation, located three residues from D240, a member of the catalytic triad, replaced a highly conserved aromatic residue in the RISF in β strand 1 with a basic amino acid and completely eliminated transposition without affecting the global binding proficiency. This substitution replaced a residue that is probably involved in positioning the DNA in the catalytic pocket [82], a change that did not affect the integrity of the β strand (see Discussion).

### The middle interval

The V179L (GMF 101) substitution occurred in α helix M5 (Figure 10a). This change disrupted binding (Figure 6f, lane 5) and completely eliminated transposition (Table 2, row 26), a result which suggests that at least α helices M4-M6 of the middle region of the protein, which are aligned with the first three N-terminal helices of the integrase protein (IN), contribute to the overall structural and functional architecture needed to facilitate binding by the protein.

## Discussion

### Rationale for soluble expression of the GFP-tagged IS*2 *TPase

GFP has been used widely as a reporter or biological marker [83], extensively in fusion constructs to determine the extent of solubility of target proteins, in protein folding assays and in directed evolution [44,84]. Although its use as an agent to facilitate the soluble expression of proteins that misfold or aggregate when overproduced in *Escherichia coli *has been approached with caution [85], success has been reported for a plant actin [86]. We reasoned that, given its robust solubility, it might be used to facilitate soluble expression of the intractably insoluble IS*2 *TPase under native conditions.

### The full length fusion protein achieves very efficient binding to cognate DNA sequences

The inefficient binding to cognate DNA of full length native or GFP-tagged IS*2 *TPase, purified to homogeneity, contrasts starkly with the extremely efficient binding of the partially purified OrfAB-GFP utilizing residues at both the N- and C-termini of the TPase. In addition, footprinting studies reported elsewhere show that the protein binds to both the protein binding and catalytic domains of IRR, generating fully formed complexes (Lewis *et al*, Protein-DNA interactions define the mechanistic aspects of circle formation and insertion reactions in IS*2 *transposition, submitted). In this study we have not explored in detail the reasons for this difference but reports of inefficient binding of full length TPases of insertion sequences are not uncommon. For example, in IS*911 *[8,15] and in IS*30 *[28,29], both of which transpose via the two-step circle-forming pathway, successful footprinting studies have only been conducted with truncated versions of the Tpase, which retain the DNA binding domain and lack the C-terminus. Inefficient binding was initially also reported for IS*50 *[87,88] and in both IS*50 *[88] and IS*911 *[15] it has been proposed that this is due to interference of binding domain function by the C-terminus. Recently, a full length calmodulin-binding peptide fusion derivative of the IS*256 *TPase, which catalyzes circle formation in this element [12], was shown to bind to the ends, but it did so much less efficiently than N-terminal fragments containing the DNA binding domain, lending additional support to this hypothesis [89]. Other reports of inefficient binding by recombinant TPases in both prokaryotic and eukaryotic transposons, such as IS*903 *[90], Tc1 [91] and TAG1 [92], has led to the speculation that improper folding during the purification process may be the cause of inefficient binding. Our results with the partially purified IS*2 *TPase suggest that an unidentified component or, speculatively, even the presence of unspecific or IR DNA may be the agent which facilitates and/or maintains proper folding in these TPases.

### The DNA binding domain of IS*2*OrfAB consists of a three-helix bundle with a defined HTH motif

The location of three α helices, which might comprise the binding domain of the IS*2 *TPase at positions 13 to 26, 32 to 38 and 43 to 55, by the PHD secondary structure algorithm of PBIL [55] represents the best fit of our data (compare Figures 9b and 11). The only discrepancy is our decision to include residues 56 to 58 in helix 3 because substitutions S57G and L58I both negatively impact binding and transposition. L58I substitutes a residue whose most pronounced effect is its difficulty in adapting to an α helix conformation because of its branched β carbon for one which shows a distinct preference for being in α helices [93]. The absence of complex formation (Figure 6c, lane 2) suggests that the substitution destabilized the α helix and likely the entire binding domain. We discuss the role that S57 plays in the recognition helix of an HTH motif below. These two substitutions suggest that residues 57 and 58 are within helix 3 or, less likely (given the potential role of S57 described below), are required for the stabilization of the helix. The R13H substitution completely abolished both binding and transposition (Figure 6a, lane 9) by replacing a polar, hydrophilic, positively charged residue that often has a structural role [94] with one which is less likely to carry a charge, making it likely that helix 1 plays an important role in the structural architecture responsible for binding the cognate DNA sequence in IS*2*. These data suggest that the binding domain includes all three helices and is comprised of residues 13 to 58 (Figure 11).

The HTH motif predicted by the HTH secondary structure analysis protocol of PBIL [54] also represents an excellent fit with our data. The motif includes residues M30 to K51 and is associated with helices 2 and 3 of the putative binding domain (compare Figures 9c and 11). The consensus sequence of Pabo and Sauer [95] which generally characterizes the HTH motif in prokaryotes supports the claim that it resides in helices 2 and 3 (Figure 11). When this consensus sequence [h_o_-G/A-(X)_2_]_helix 1-_[h_o_-G-h_o_-X]_turn_-[(X)_3_-I/L/V-...]_helix2_, is applied to residues M30 to L58, (where h_o _is a hydrophobic residue, and × is any residue) we see a very reasonable fit: [**V35**-**A36**-R37-Q38]_helix 1_-[**H39**-**G40**-**V41**-A42]_turn_-[A43-S44-Q45-**L46**....]_helix2_. The critical residues here (in bold) are, (i) the optional hydrophobics (h_o_), V35 in helix 1 and H39 and V41 in the turn (histidine has the potential to be buried like a hydrophobic [93]) and (ii) three conserved hydrophobics, A36 in helix 1, the invariant glycine (G40) in the second position of the turn (both weak hydrophobics) and L46 in helix 3 (Figure 11).

It is interesting that four of the nine randomly induced substitutions in the binding domain affected residues in this consensus sequence. A comparison of the effects of the S44N substitution and of the R37Q/S44N double replacement in helices 1 and 2 respectively of the proposed HTH motif gives some additional insight into the role of these two residues in the stabilization of the HTH motif. Since the drastic effect of S44N (no detectable binding and 80-85% reduction in the transposition frequency, Figure 6a, lane 7) is partially reversed by R37Q/S44N (about 60% and 65% reduction in binding and transposition frequency, respectively, Figure 6b, lane 2), we make the following assumptions: S44 and R37 are likely involved in interhelix H-bonding and contribute to stabilizing the HTH. In the S44N mutant derivative, arginine and asparagine are apparently not as effective in H-bonding, resulting in a destabilized motif. H-bonding by glutamine and asparagine in the double mutant, however, appears to be partially restored, most likely because of the increased capacity of this pair of amino acids to form H-bonds [94].

The fact that four of the seven mutations which disrupted binding occurred in the second helix of this HTH motif (Figure 11) supports the convention that it is the recognition helix. Two of these substitutions, R50H and S57G, help identify residues that are likely involved in making specific DNA contacts. The R50H substitution in the putative recognition helix produced a protein derivative which generated the partially dissociated complex in Figure 6a, lane 5 and completely eliminated transposition. In this case the positively charged arginine is replaced by an amino acid whose flexibility in shedding its proton allows it to readily assume a neutral state, making it less effective as a residue involved in binding to DNA sequences [93] and suggesting that R50 plays a pivotal role in recognizing its cognate DNA sequence. Because the IS*2 *transposition pathway requires separate binding events for each of the two steps, even a moderate reduction in binding would probably have a drastic effect in reducing transposition frequency, as seen with R50H. S57G substitutes a small residue without a side chain for a polar hydrophilic residue with a fairly reactive OH group, which is usually involved in forming hydrogen bonds. Since this residue is located in the putative recognition helix, a DNA-contact assignment to S57 could also explain the effect of this substitution in generating the dissociated complex in Figure 6c, lane 2.

Two substitutions, A42T and V35L, which produced little or no change in the wild type phenotype, lend additional support to our identification of the HTH based on the Pabo and Sauer predictions. Replacement of A42 in the four-residue turn with any small amino acid would probably have little effect on protein function (A42T; Figure 6c, lane 3); in addition, the replacement of the optional hydrophobic, V35, with leucine in the first helix of the HTH would not be expected to have a significantly negative effect (Figure 6b, lane 5) on HTH function (see Figure 11). These results confirm that in IS*2*, N-terminal helices 2 and 3 contain the HTH motif with a four-residue turn between them. Thus the IS*2 *binding domain consists of residues 13 to 26 which form helix 1, 32 to 38 form helix 2, (helix 1 of the HTH; Figure 11), 39 to 42 form the turn, and 43 to 58 form helix 3 (helix 2 of the HTH; Figure 11). The A42T mutation has an interesting phenotype in that it was selected as a bright colony (see the legend to Table 3) but is not toxic to the cell even though it is phenotypically a silent mutation. It is possible that its protein is produced in lower amounts or that the mutation has simply made the protein more soluble.

These results are in general accord with, and extend the work of, Prere *et al. *[52], Hu *et al. *[5], Lei and Hu [33] and Rousseau *et al. *[34] on IS*3 *family TPases. Hu *et al*. predicted the existence of an HTH motif in the IS*2 *TPase at residues 31 to 50 and Lei and Hu demonstrated the loss of binding capability experimentally for IS*2 *OrfA deletion derivatives lacking as few as the first 12 residues (likely destabilizing the formation of helix 1) and as many as 57 residues from the N-terminus. PSIPRED secondary structure analyses of the TPases of all other prototypes of the principal subgroups of the IS*3 *family show three helices whose positions are similar to those shown for IS*2 *(data not shown).

There is much evidence for multihelix binding domains which include at least one HTH motif in TPases. IS*30*, which transposes via a circle-forming pathway, possesses an N-terminal binding domain with two HTH motifs, one of which is a component of an H + HTH structure [28]. The MuA Iβ and Iγ DNA-binding subdomains which form bipartite binding structures are composed of five and four α helices, respectively, each including an HTH motif [96,97]. In the case of the Iβ subdomain of MuA, all five helices are involved in the interaction with the DNA. Similar results have been reported for the TPases Tc3 [98] and the Tc1-like element *Sleeping Beauty *[99] whose multihelix structures with two HTH motifs are not dissimilar from those of the homeodomain family of helix-turn-helix DNA-binding proteins [100] or the *paired *DNA binding domain family [101].

The W49R substitution in the second and putative recognition helix of the HTH generated a protein with no negative effects on binding efficiency (Figure 6b, lane 3) but lacked any capacity for transposition (Table 2, row 11). Resolution of this apparent contradiction has led to the conclusion that W49 may not directly interact with the protein binding domains of IRR and IRL. Figure 7 shows that few residues in the N-terminal helix 3 (B α-3) in IS*2*, are conserved in IS*3 *family TPases. This is expected for the recognition helices of these motifs which have little identity in the sequences of their ends; on the contrary, W49 in IS*2 *however, corresponds to what has been described as one of the most highly conserved of all residues in the TPases of the IS*3 *family [34]. The ability of the W49R mutation to disrupt transposition but not binding in IS*2*, (even when a charged hydrophilic residue is substituted for a highly hydrophobic one) suggests that the function of W49 may extend globally in the protein and is not confined to binding functions of the HTH motif.

A similar but not identical inconsistency in the relationship between binding efficiency and transposition was also observed with the equivalent W42 in IS*911 *[34]. There, the W42F mutant derivative which produced little to no binding efficiency with a truncated OrfAB lacking the CAS, showed a strongly positive result for *in vivo *transposition in the presence of the CAS of the IS*911 *TPase. This suggested that the CAS somehow had the ability to compensate for the deficiency of the W42F substitution in facilitating binding.

Our results suggest that this conserved tryptophan in IS*3 *family TPases may be involved in interacting with the CAS of the protein, for example, by promoting the folding which allows that motif to be correctly positioned in binding to the catalytic domain of IRR. W49R may fail to communicate the level of accuracy in CAS binding (for example, by permitting a minor misfolding) that is needed to allow recombination, without affecting regional DNA binding. Evidence for extensive binding of the IS*2 *TPase to the catalytic domain of IRR (the donor end in this insertion sequence) has been shown in concurrent footprinting studies described elsewhere (Lewis *et al*, Protein-DNA interactions define the mechanistic aspects of circle formation and insertion reactions in IS*2 *transposition, submitted) and the issue of the role of the CAS in global binding of the protein is addressed in this study in the discussion of CAS mutations which reduce binding efficiency.

### The IS*2 *TPase possesses a LZ-like oligomerization motif at its N-terminus that facilitates binding to the ends of the element

The sequence of the coiled coil motif of the IS*2 *OrfAB TPase (residues 73-100; Figure 12a) differs in significant ways from that of the canonical LZ. Indeed when this sequence is tested on the 2-ZIP server (2zip.molgen.mpg.de/cgi-bin/2zip.pl;[59]) a LZ is not predicted. In this study, all five substitutions in the coiled coil domain indicate that a LZ-like motif, whose function is required for binding and transposition, exists within residues 73 to 100 in the IS*2 *TPase.

We have aligned the four OrfAB LZ-like heptads in IS*2 *with corresponding sequences from prototype elements of the four other subgroups of the IS*3 *family (Figure 12b). Haren *et al. *[30] have, however, created a detailed alignment of putative LZ sequences from OrfA, involving 15 members of the five subgroups (IS*2*, IS*3*, IS*51*, IS*150 *and IS*407*) of the IS*3 *family and they have specifically demonstrated the presence of a canonical LZ motif with a four-heptad repeat in OrfAB of IS*911 *[30,31]. These alignments reveal, however, that the putative IS*2 *LZ-like motif is the only sequence in which only two of the four **d **positions are occupied by leucine (L83 and L97) and that IS*2 *alone lacks the leucine residue at the **d **position of the first heptad (for example, see A76-; Figure 12b). However, three of the four hydrophobic residues at the **a **positions (L73, I80 and L87) are occupied by leucines or isoleucine. The fourth **a **position, N94, in the fourth heptad is the buried polar asparagine, which is essential for inter-subunit H-bonding in canonical LZ structures [102]. Another significant difference between this putative IS*2 *LZ-like motif and the canonical LZ is the restriction of ionic (**g**/**e**' **g**'/**e**) stabilizing salt bridges to the third and fourth heptads (Figure 12c). It is possible, however, that weak non-ionic inter-subunit stabilizing interactions between the first and second heptads are brought about by the glutamine residues (Q79 and Q84) in the **g **and **e **positions of these two heptads. We propose, based on the analysis of all five mutations, that stabilization of a potential LZ-like structure (Figure 12c) would be brought about as follows: the N-terminal half of the structure would be relatively weakly stabilized by the concerted action of the **d**-located leucines at L83 in the second heptad, the **a**-located hydrophobics L73 and I80 and by hydrogen bonds at the **g **and **e **positions, Q79 and Q84, in the first and second heptads respectively. The C-terminal half of the motif, on the other hand would be more strongly stabilized by the **d**-located leucines at L97, the **a**-located asparagine (N94) whose buried hydrogen bonds contribute significantly to stabilization of the zipper (both in the fourth heptad) and the canonical ionic salt bridges generated by the **g **and **e **residues at E93 and K98 in the third and fourth heptads, respectively. Thus, L83V and L97H affected the canonical **d**-located leucines. The L83V substitution (Figure 6c, lane 5) completely abolished both binding and transposition, suggesting that substitution of the C-β branched valine residue destroyed the primary interaction for stabilization at the N-terminus and consequently the entire LZ-like motif. The phenotype of the Q79L substitution appears to have affected the weak **g**/**e**' **g**'/**e **inter-subunit stabilizing reactions at the N-terminal end of the zipper-like structure but, given that the primary stabilization interaction is still present, it produced a less drastic phenotypic change insofar as binding efficiency is concerned (Figure 6a, lane 4), compared to the replacement at Leu^83 ^(L83V).

L97H, on the other hand, had a much less drastic effect on binding (Figure 6a, lane 6), although transposition was all but abolished. The L97H substitution destabilized the putative motif at its C-terminal end but the two other strong stabilization interactions described above appear to allow a level of oligomerization that permits unstable binding with minimal dissociation. Similarly, N94D altered the buried **a**-located asparagine residue required for stabilization of the zipper but the existence of the two remaining stabilization interactions at the C-terminus appears to have influenced the production of a phenotype similar to that of L97H (Figure 6c, lane 5).

The K89M substitution (Figure 12c) also abolished transposition completely and provides further evidence for a functional LZ-like motif. Its phenotype is consistent with the location of K89 at a **c**-located position, which is part of the solvent-exposed helical surface that must be occupied by a hydrophilic residue. A hydrophobic residue would disrupt the formation of that surface and subsequently abolish zipper function [103,104].

### The CAS of the TPase of IS*2 *and other IS*3 *family members share the functional properties of the three-dimensional catalytic core of the TPase/RISF

The eight substitutions, W237R, L266P, H267D, R291H, V301M, A341T, A341P and E391K (Table 2, rows 18-25) fell into 3 α helices and 3 β strands of the putative CAS (Figure 10b). Four of these (W237R, L266P, H267D and V301M) impacted the putative β sheet of the catalytic core and abolished transposition but only W237R had no effect on binding (Figure 6f, lane 4), a result that helps identify the function of W237 and of β strand 1 in the CAS. Two of the remaining four mutations, A341T and A341P, located adjacent to the third member of the catalytic triad, E342, affected a highly conserved hydrophobic residue in α helix 4 in the RISF, that is, V151 in HIV-1 (Figure 10b; see also [105]). A341T had no negative effect on binding efficiency (Figure 6b, lane 4) and enhanced the frequency of transposition by about 50% (Table 2, row 22), a result that also sheds light on the function of α helix 4 in the IS*2 *CAS. Substitutions were recovered in two other α helices, E391K in α helix 6 and R291H in α helix 1. These and H267D in β strand 3, which reduced but did not eliminate binding, helped identify residues and elements which likely function in binding the CAS to the catalytic domain.

The W237R and A341T substitutions eliminated and enhanced cleavage respectively, and provide strong evidence, based on the deduced function of the two WT residues, that the three-dimensional structure of the catalytic core of the IS*2 *TPase functions similarly to that in the RISF. W237R is highly conserved in β strand 1 of the RISF and aligns with W61 in HIV-1 and RSV. The location of this tryptophan, three residues from the first of the catalytic aspartates (D240 in IS2 and D 64 in HIV-1) on β strand 1, is consistent with its role, as shown from crosslinking studies with W61 of HIV-1 [106], in interacting with the 3' end of the DNA and positioning it within the catalytic pocket. The ability of W237R to eliminate transposition without affecting binding could then be explained by a similar role for W237.

The A341T substitution highlights the essential supporting role of residues adjacent to E342 in α helix 4, in the chemistry of cleavage and joining, and we draw this conclusion from the extent of conservation in this α helix in the RISF. For example, the co-crystal structure of the Tn*5 *TPase has shown that Y319, R322, K330 and K333, which flank E326 (the triad glutamic acid) in α helix 4, are involved in making specific contacts with the 3' and 5' ends (transferred and non-transferred strands) of the catalytic domain of the DNA [67]. These four residues are aligned directly, in α helix 4 of IS*2*, with E336, N338, K346 and K349 (N338 and K349 are highly conserved residues), which flank E342 [61] and presumably have the same function as their equivalents in Tn*5*. In addition, K346 and the conserved K349 in IS*2 *are aligned with K156 and K159 in HIV-1 integrase (Figure 10b). These two residues in IN have been shown to contact the DNA, with K159 directly interacting with the adenosine of the terminal CA-3' dinucleotide, where it is involved in orienting the DNA properly for cleavage [83]. Earlier, van Gent *et al. *[107] had shown that a K159V substitution in HIV-1 significantly slowed the rate of integration without significantly reducing the amount of integration in an overnight incubation. Their implication was that this mutation reduced by one the number of residues flanking E152 (the triad glutamic acid) available for contact with the DNA and thus reduced the efficiency of interaction between the protein and the DNA. In addition, Calmels *et al. *[108] demonstrated in HIV-1 that 75% of the random mutations immediately flanking E152 that resulted in an increase in the amount of binding to a strand transfer substrate included a V151T mutation, the homologue of A341T in IS*2*. One can then account for the 50% increase in transposition of A341T, by assuming that enhanced interaction with the catalytic domain of IRR, due to an additional specific or stochastic DNA contact by the substituted threonine, produced the subsequent enhancement. This is likely the case, given its proximity to the four residues which putatively make contact with the catalytic domain of the IS*2 *IRR and its location adjacent to E342. These two results, with W237R and A341T on β strand 1 and α helix 4 respectively, suggest that the three-dimensional structures of these elements, and subsequently that of the catalytic core, are functionally similar to those of the RISF.

We have been able to differentiate between substitutions in the CAS which do not affect the binding efficiency of the protein, W237R or A341T, those which affected the structural integrity of the catalytic core and thus the entire protein, preventing any complex formation, A341P, L266P and V301M, (Figure 6e, lanes 2-4) and those which reduce binding efficiency of the CAS to the cognate DNA, such as H267D, R291H and E391K (Figure 6a, lane 1 and 6d lanes 2-3); these last three produced partially dissociated complexes identifying residues that are likely important binding contacts between the CAS and the catalytic domain. H267D replaced a basic residue with a negatively charged one at a non-conserved position on β strand 3. The enhanced level of substrate dissociation is in accord with reduced contact with the DNA. R291H substituted a weakly basic residue at a position occupied by a conserved arginine in four of the five subgroups in α helix 1 of the IS*3 *family. The substitution reduced binding efficiency, likely compromising the DNA anchoring function provided by Arg 291. E391K occurs in α helix 6, which is characterized by two highly conserved residues, proline (P389 in IS*2*) in RSV and the IS*3 *family and a glutamic acid or glutamine in the RISF; E391K in IS*2 *altered the latter and the replacement of the acidic residue with the basic lysine reduced the overall binding affinity to the DNA in the catalytic domain, without completely eliminating it. The phenotypes of these mutations (H267D, R291H and E391K) suggest that their wild type residues are critical contacts which facilitate the binding of the CAS to the catalytic domain of IRR.

On the other hand, A341P, the helix-breaking proline substitution in α helix 4, altered a conserved hydrophobic residue in the RISF, significantly reducing complex formation. L266P altered a conserved hydrophobic residue in β strand 3 of the RISF and V301M altered a very hydrophobic, conserved residue in the IS*3 *family in β strand 4, associated with the second aspartate of the catalytic triad (D306); both of these completely eliminated complex formation. The fact that all three of these substitutions replaced very hydrophobic residues and eliminated binding suggests that their principal effect was to disrupt the α helix or β strand, or the putative β sheet and thus the catalytic core, the integrity of which is clearly essential for proper folding of the full length protein and thus global binding.

These results underscore the importance that binding of the catalytic core to the CD plays in regional and global binding of the full length protein. On one level the W49R substitution in the recognition helix of the HTH apparently failed to coordinate the necessary level of accuracy of binding of the catalytic core to the DNA of the catalytic domain (most likely due to a minor folding impairment), eliminating transposition but nevertheless permitting global binding. However, a full length protein with a mutation of a single anchoring residue in its catalytic core, which may not alter the structural integrity of the protein, significantly impacts global binding, manifested by partial dissociation of the complex. From this we conclude that the binding reactions with wild type proteins shown in Figures 2 and 6, in which all of the DNA is driven into the complex, result from fully formed complexes in which both the DNA binding domain and the CAS of the protein are fully complexed to the ends. This conclusion is supported by data showing extensive protection of the protein binding and catalytic domains of IRR or of the abutted ends of the minicircle junction (Lewis *et al*, Protein-DNA interactions define the mechanistic aspects of circle formation and insertion reactions in IS*2 *transposition, submitted). Impaired binding by either domain of the protein thus produces dissociation of the complex.

### The integrity of a middle interval contributes to the binding capability of the IS*2 *TPase

The V179L substitution affects a hydrophobic residue that is functionally conserved in α helix M5 in the RISF (Figure 10a). Two of the three residues conserved in the IS*3 *family are also conserved in the RISF and V179L affected one of them. The disruption of binding and abolition of transposition in IS*2 *likely resulted from the replacement of the C-β branched valine, which affected the backbone of the α helix, distorting or disrupting it [93]. The result suggests that at least α helices M4 to M6 of the middle interval of the protein, which align with good conservation with the first three α helices of IN, are critical to the functional architecture of the protein that relates to global binding to the cognate IS*2 *DNA.

## Conclusions

These results validate the strategy of the GFP-tagged approach to obtaining, under native conditions, preparations of a full length, soluble, active protein like the IS*2 *TPase that is usually insoluble when prepared under native conditions and refractory to whole protein structure-function or biophysical studies when solubilized. This strategy has resulted, for the first time (among circle forming insertion sequences with a two-step transposition pathway), in the recovery of a full length protein which is capable of very efficient binding *in vitro *to cognate DNA and the formation of fully formed complexes (Lewis *et al*, Protein-DNA interactions define the mechanistic aspects of circle formation and insertion reactions in IS*2 *transposition, submitted) involving residues at both the N- and C-termini of TPase. In addition the fluorescence-based random mutagenesis approach to exploring structure-function relationships has helped refine our understanding of those relationships in IS*2 *and the IS*3 *family TPases by teasing out residues that facilitate binding, oligomerization and (as they relate to the integrases) catalysis, as well as those that define possible interactions between structural motifs of the protein.

## Methods

### Bacterial strains and media

*E. coli *strain JM105 (New England Biolabs) was used for most procedures involving plasmid DNA preparation, cloning and the *lacZ *papillation assay. DNA transformation was carried out into supercompetent XL1 Blue cells (Stratagene Inc, Santa Clara, CA, USA) for reactions requiring cloning and overexpression of the fused *orfAB *and *GFPuv *genes in pLL2522. BL21(DE3)pLysS cells (Novagen-EMD4Biosciences, La Jolla, CA, USA) were used for over expression of the OrfAB-GFP fusion product cloned into the pTWIN2 vector (New England Biolabs).

Cultures were routinely grown in lysogeny broth (LB) media at 37°C, supplemented where necessary with carbenicillin (Cb, 50 μg/mL), kanamycin (Km, 40 μg/mL) or chloramphenicol (Cm, 20 μg/mL). For the overexpression of pGLO, pLL2522 and pLL2524-XXX (plasmids with the GMF mutations), cultures were grown at 28°C in 2x YT media supplemented with Cb and arabinose (6 mg/mL).

### DNA procedures

Plasmid DNA preparation was carried out using the standard alkaline lysis procedure of the Wizard DNA Purification System (Promega Corp., Madison, WI, USA) for in-labarotory protocols. The Pure Link HQ Miniplasmid Purification Kit (Invitrogen Corp., Carlsbad, CA, USA) was used in the preparation of DNA samples for outsourced sequencing reactions (see below).

Restriction endonuclease digestion was carried out with enzymes and buffers from New England Biolabs. Diagnostic gels were made with 0.8% Seakem agarose and preparative gels were made with 0.6% Seaplaque Low Melting Temperature agarose (Cambrex Corp., East Rutherford, NJ, USA). DNA was purified from preparative gels with Gelase (Epicentre Biotechnologies, Madison, WI, USA) following the manufacturer's instructions and concentrated in a Microcon-100 Filter Device (Millipore, Billerica, MA, USA) to a 50 μL volume. The solution was dried down to a pellet in a Savant SpeedVac DNA concentrator, resuspended in 12 μL ultrapure H_2_O and frozen at -20°C until use. Standard cloning procedures were as previously described [7].

Standard PCR and PCR-mediated *in vitro *site-directed mutagenesis were carried out with the Vent DNA polymerase (New England Biolabs) used in accordance with the manufacturer's instructions. The reaction protocols were as described earlier [6]. PCR products were cleaned up with the Direct PCR Purification Buffer and the Wizard PCR Preps Resin (Promega Corp.).

### Plasmid constructs and mutagenizing oligonucleotides

pLL2522 which contained the fused *orfAB *and *GFPuv *genes (Figure 2e) was prepared following the procedure illustrated in Figure 2.

pGLO-ATG2 containing 3'-located *Eco*RI-*Nhe*I cloning sites (Figure 2a) was created by removing an *Eco*RI site located adjacent to the two stop codons (bold upper case) at the 3' end of *GFPuv *with the oligonucleotide (all mutagenizing sites in this section are in bold lower case) 5'GGATCATCAGGTACCGAGC**g**CG**t**AT**TCATTA**TTTGTAGAGCTCATCCATGCC3' and creating a new cassetting *Eco*R1 site upstream of the existing *Nhe*I site (in upper case, containing the first two codons of GFP) and destroying the ATG start codon at the 5' end of the gene, with the oligonucleotide 5'TCCCCTTCCCC**GCTATGg**ATCAGCTGA**gaattc**TTCTCCTTCTTAAAGTTAAA3'.

pLL2521HK (Figure 2d) containing an *Eco*RI-*Nhe*I cassetted *orfAB *gene was created in successive steps by removing the upstream *Eco*RI site in pLL18 (Figure 2b) with the oligonucleotide 5'AGACTATCACTTATCCGCGGAACAGTCTAGAGCTC**cccctc**ACTGGCCGTC3', placing *Eco*RI adjacent to the IS*2 *start codon (pLL2509A; Figure 2c) with the oligonuclotide 5'ACTAGTTTTTAGACCGTCATTGGA**gaattc**ATGATTGATGTGTTAGGGCC3', adding an *Nhe*I site and altering the adjacent stop codon at the 3' end of IS*2 orfAB *to create pLL2520 with the oligonucleotide 5'GGGCCC**gcgctagc**ACCGGTTATTTCCAGACATCTGTTATCACTTAACC3' and adding a 6X HIS tag downstream of the IS*2 orfAB *start codon (Figure 2d) with oligonucleotide 5'GTATG**catcatcatcatcatcatagcagatatctggtattgagtataagc**ATTGATGTCTAAGGGCCGGAG3'Finally, in order to fuse the *Eco*R I-*Kpn*I cassetted *orfAB-GFPuv *fusion sequence (Figure 2e) to the Km^r ^reporter gene, a procedure needed for the creation of *lacZ *papillation assay constructs, a *Kpn*I site was added adjacent to and downstream of the *Nhe*I site (upper case lettering) in the sequence that connects *orfAB *to the Km^r ^gene. For this we used the primer 5'AACTGATCCAGGGCCCG**ggtacc**A**GCTAGC**ACCAGTTATTTC3'.

pLL2522 was produced by cloning the cassetted *Eco*R1-*Nhe*I *orfAB *gene into pGLO-ATG2 (Figure 2e).

pUH2509, a construct used for *lacZ *papillation assays, containing IS*2 *with the frame fused *orfAB *gene from pLL18 (Figure 2b) was created as follows. IRL in pLL18 was deleted and the weak indigenous E-10 promoter (upper case lettering) conserved while adding a *Sac*II site to form pLL2509A (Figure 2c), into which the *Xba*I-*Sac*II cassetted *lacZ *gene could be cloned. We used the oligonucleotide 5'CCAGTGGAATTCGAGCTCTAGACTGTT**ccgcgg**ATAAG**TGATAGTCT**TAATATTAGTTTTTTAGACTAGTCATTGG3'. *lacZ *was obtained from pLL135 [19]. The 3' end of the gene was modified to add the necessary *Sac*II site, generating pLL135II using 5'GGTACCGGGGATCC**gccg**AGACATGATAAGATACATTGATGAGTTTGG3'. The 5' end of *lacZ *was modified to remove the lacUV5 promoter, to add an *Xba*I site as well as the IS*2 *IRL (upper case lettering) generating LL135IRLLZ. All three reading frames reading into the IRL sequence lacked stop codons. We used the oligonucleotide 5'ATGTTCTTTCCTCGAG**tctaga**TAGACTGGCCCCC**TGAATCTCCAGACAACCAATATCACTTAATTAT**TGCCGTAAGCCGTGGCCG3'. The *Xba*I-*Sac*II fragment from pLL135IRLLZ was cloned into pLL2509A to produce plasmid pUH2509, which contained a 6.4 kb version of IS*2 *consisting of (from 5' to 3'): IRL, the promoterless *lacZ *gene sequence, the *orfAB *sequence without functional left or right ends, the Km^r ^gene and IRR.

pUH2523, the construct containing the fused *orfAB::GFPuv *genes, used for *lacZ *papillation assays, was created as follows. (i) *orfAB *linked to the Km^r ^gene in pLL2521HK is cassetted within *Eco*RI and *Kpn*I restrictions sites (Figure 2d), so in order to add the Km^r ^reporter gene to the fused *orfAB::GFP *genes we replaced *orfAB *in pLL2521HK (Figure 2d) with the *Eco*RI-*Kpn*I cassetted *orfAB::GFP *sequence shown in Figure 2e, to create pLL2523. (ii) The *lacZ *papillation assay plasmid pUH2509 possesses an *Spe*I site downstream of the E-10 promoter of IS*2orfAB *and an *Nru*I site within the Km^r ^reporter gene, as do all constructs in which Km^r ^is present as a reporter gene (see, for example pLL2521HK in Figure 2d). The *Spe*I-*Nru*I fragment from pUH2509 was replaced by the corresponding fragment from pLL2523 to create pUH2523. Similarly, *Spe*I-*Nru*I fragments from pLL2524-XXX, plasmids containing mutated *orfAB *genes (see below), were used to create *lacZ *papillation plasmids pUH2524-XXX.

pUH2523Δ*orfAB*, the null mutation used as a control in *lacZ *papillation assays (Table 2, row 1), was created by deleting a 1743 bp fragment between two *Mfe*I restriction sites, 103 bp from the start of the IS2*orfAB *sequence and 156 bp from the end of the *GFPuv *sequence in pUH2523, followed by blunt ligation of the sites.

pTW2*orfAB::GFP *was created by cloning the fused *orfAB::GFP *genes into the pTWIN2 vector of the IMPACT system (Intein Mediated Purification with an Affinity Chitin-binding Tag; New England Biolabs) for the purposes of improving the purification of the fusion protein. The construct was cloned into the N-terminal multiple cloning site of the vector by first creating a *Sbf*I site close to the existing *Eco*RI site with 5'GGCATACATGAATTCCTCGAGG**cctgcagg**CTGCGTATCCGGTGACACC3' to accommodate the *EcoR*I/*Sbf*I cassetted *orfAB::GFP *sequence.

### Creation and cloning of mutations in IS*2 orfAB *from a PCR-based random mutagenesis protocol

The GeneMorph II Random Mutagenesis Kit (Stratagene) was used to create mutations within *orfAB *in pLL2521HK (Figure 2d) using a 30-cycle PCR-based protocol. Primers were M13F (forward) and KmR1 (reverse; [6]). Mutations were generated at very low, low and medium rates (900 ng of target DNA within 3.6 μg of plasmid DNA; 500 ng of target within 2.0 μg of plasmid DNA; and 250 ng of target within 1.0 μg of plasmid DNA respectively). PCR products were cloned into the *Eco*RI-*Nhe*I sites of pGLO-ATG2, transformed into XL1-Blue Supercompetent cells and plated onto LB plus Cb plus arabinose agar. After 72 hours at 37°C, plates were examined for brightly fluorescing colonies among a background of less brightly fluorescing colonies. Plasmids from the brighter fluorescing clones carrying mutations in the *orfAB *sequence were identified as pLL2524-XXX where XXX stands for 001-110.

### *LacZ *papillation assays

Papillation was best observed when pUH2509, pUH2523 or pUH2524-XXX plasmid DNA was transformed into JM105 cells. The DNA concentration was titrated to produce about 50 to 60 transformants per plating on to LB plus Km plus Cb plus arabinose agar. Plates were incubated in airtight bags to minimize drying. The numbers of papillae plateaued after 20 to 25 days at 37°C.

### Preparation of the wild type and mutant OrfAB-GFP fusion proteins under native conditions

pLL2522 and other mutant plasmid DNA were transformed into XLI-Blue cells (Stratagene), plated on to LB plus Cb plus arabinose agar and incubated for 48 hours at 37°C. A single fluorescing colony was inoculated into 10.0 mL of similarly supplemented 2x YT broth and incubated overnight at 28°C. After centrifugation, the pellet was checked for fluorescence, washed in 3.0 mL Native Wash Buffer pH 8.0 (50 mM sodium phosphate monobasic monohydrate, 300 mM NaCl), resuspended in 3.0 mL Bug Buster Protein Extraction Reagent (Novagen-EMD4Biosciences) supplemented with 1.0 uL of Benzonase (Novagen-EMD4Biosciences) per 10.0 mL overnight (o/n) culture and 3.0 uL of Protease Arrest (Calbiochem-EMD4Biosciences La Jolla, CA, USA) per mL of lysate and nutated at 4°C for 30 minutes. If necessary, the suspension was subjected to a single round of freezing and thawing to complete lysis. The lysate was checked for bright fluorescence before and after centrifugation at 16,000 × g for 1 hour at 4°C.

6xHis-tag purification of the protein was achieved by gravity flow affinity chromatography using Ni-NTA agarose (Qiagen Valencia, CA, USA) under native conditions essentially following the manufacturer's instructions. The crude lysate was loaded on to a 1.0 mL bed of the nickel-charged resin in a 5.0 mL column and chromatographic separation followed with UV light. The protein bound as a tight brightly fluorescing band at the top of the column and remained bound through washings with 10 to 60 mM Imidazole when a slight dissociation of the band was observed. To circumvent continued dissociation, the band was eluted with 250 mM Imidazole and its progress through the column followed. Peak fractions (fluorometrically determined) were subjected to diagnostic 12% PAGE using Ac:Bis (30%:8%) polyacrylamide gels (Figure 4a). Fractions showing both the 74 kDa OrfAB-GFP and the 17 kDa OrfA proteins were pooled (approximately 700 uL), concentrated to about 75 uL in a YM-10 Microcon Centrifugal Filter Device (Millipore), dialyzed overnight in 300 mM NaCl, 50 mM tris(hydroxymethyl)amino methane (Tris-Cl), pH 8.0 and 1.5 mM dithiothreitol using Slide-A-Lyzer cassettes (Pierce/Thermo Scientific Rockford, IL, USA) and stored in 50% glycerol at -20°C. Concentrations of GFP in the sample shown in Figure 4a were measured with spectrophotometry at 280 nm and 397 nm while those of the wild type and mutant versions of the fused OrfAB-GFP proteins were measured at 397 nm. Comparative levels of fluorescence of GFP and the fusion proteins were measured fluorometrically and used to confirm the concentration data.

For the overexpression of the OrfAB-GFP fusion protein in the pTWIN2 derivative (IMPACT, New England Biolabs), plasmid pTW*orfAB::GFP *was transformed into BL21(DE3)pLysS cells. Single colonies were inoculated into 10 mL 2xYT plus Cb plus Cm and grown overnight at 37°C. Two milliliters of this starter culture was inoculated into 120 mL of the same medium (to establish an optical density (OD) of 0.2) and grown at 37°C to an OD of 0.8 when it was induced with 1.0 mM isopropyl β-D-1-thiogalactopyranoside and allowed to grow overnight at 16°C. The culture was lysed as described above and the cleared lysate loaded onto the chitin column. The protein was purified per the manufacturer's instructions with binding and elution monitored by UV light-induced fluorescence. Peak fractions were collected pooled and analyzed as described above, purified on ion exchange Q-sepharose columns (HiTrap Q XL, GE Healthcare) following the manufacturer's instructions, and concentrated, dialyzed and stored as described above.

### Electrophoretic mobility shift assays

#### Oligonucleotides used

Annealed 50-mer oligonucleotides containing the 41 bp IRR sequence were used in all but one of the EMSA experiments (Figure 6a-e). The upper strand was labeled at the 5' end with γ^32^P-ATP. Primer A - upper strand (the IRR sequence is within the square brackets): 5'GGATCC[TTAAGTGATAACAGATGTCTGGAAATATAGGGGCAAATCCA]GCG3'. Primer B - lower strand: 5'CGC[TGGATTTGCCCCTATATTTCCAGACATCTGTTATCACTTAA]GGATCC3'.

Reactions shown in Figure 6f utilized annealed 87-mer oligonucleotides containing the IRR sequence. The top strand (primer A) was labeled at its 5' end with γ^32^P-ATP. Primer A - 5'GCTGACTTGACGGGACGGGGATCC[TTAAGTGATAACAGATGTCTGGAAATATAGGGGCAAATCCA]ATCGACCTGCAGGCATATAAGC3'. Primer B - 5'GCTTATATGCCTGCAGGTCGAT[TGGATTTGCCCCTATATTTCCAGACATCTGTTATCACTTAA]GGATCCCCGTCCCGTCAAGTCAGC3'.

#### 5'-end labeling and annealing of the primers

A 20 μL labeling reaction contained 40 units of T4 polynucleotide kinase in 1X T4 polynucleotide kinase reaction buffer (New England Biolabs), 20 μM of the primer (upper strand) and 50 μCi of γ^32^P-ATP. The reaction was incubated at 37°C for 30 minutes and heat-killed at 90°C for 5 minutes. A 100-μL annealing reaction contained 10 ρmol and 13 ρmol of the labeled and unlabeled strands respectively, 20 mM Tris-Cl pH 8.0 and 100 mM NaCl. The reaction was placed in a boiling water bath, cooled to 65°C, held there for 15 minutes and allowed to cool to room temperature.

#### EMSA

Binding of the TPase to its cognate DNA was carried out for 30 minutes at room temperature (20°C) in a 15-uL reaction mixture of 20 mM Tris-Cl pH 8.0, 1 mM ethylenediaminetetraacetic acid, 5.0 μg/mL calf thymus DNA, 10 nM of the radioactively labeled annealed primers and 0.13 μM of the partially purified preparation of the OrfAB-GFP fusion protein. Reactions were separated on 5% native polyacrylamide gels at 4°C for an average of 450 volt hours (Vhrs) (see Figure 6).

### Secondary structure algorithms and protein alignment tools

The ExPASy SWISS PROT translation toolkit [49] of the Swiss Institute of Bioinformatics was used to translate DNA sequences from the prototypes of the principal subgroups of the IS*3 *family, that is, IS*2*, IS*3*, IS*51*, and IS*407 *plus IS*911 *of the IS*3 *subgroup and IS*861 *of the IS*150 *subgroup, into protein sequences. Similar translations were done for sequences of the HIV-1 and RSV integrases. The ClustalW2 multiple alignment tool [50] was used for the alignment of protein sequences in Figure 7. Structure-based alignments in Figure 10 were determined from the sequences shown in Figure 7, from published RSV and HIV-1 sequences [73,109,110] from the alignments of Fayet *et al. *[60] and Rezsohazy *et al. *[61] and from the PSIPRED secondary structure determinations for the members of the IS*3 *family sub-groups and the two integrases. In these aligned sequences, functionally conserved non-polar hydrophobic residues were identified as h1 when all sequences possessed only very hydrophobic residues (L, I, V, C, M, F or W) and h2 when less hydrophobic residues are present or the conserved residues are only found in fewer than 80% of the sequences. Three different algorithms were used for secondary structure predictions: the PSIPRED server[51], the PROF Secondary Structure Prediction Protocol [53] using the Bioinformatics Information toolkit of the Max Planck Institute for Developmental Biology and the PHD Secondary Structure Analysis Algorithm [55] from the secondary analysis prediction protocol of PBIL (pbil.univ-lyon.fr; [54]). A PCOILS algorithm for coiled coils from the Bioinformatics Information toolkit of the Max Planck Institute for Developmental Biology [57,58] was used to predict the presence of a coiled coil motif and the 2ZIP server [59] from the same institution was used to predict the presence of a LZ within the coiled coil motif.

## List of abbreviations

Cb: carbenicillin; CAS: catalytic active site; CD: catalytic domain; EMSA: electrophoretic mobility shift assay; E-10: extended-10 promoter; F-8: figure-of-eight; GFP: green fluorescent protein; IRR/IRL: right and left inverted repeats; IS: insertion sequences; IR: inverted repeat; kb: kilobases; kDa: kiloDaltons; LB: lysogeny broth; LZ: leucine zipper; MCJ: minicircle junction; NaCl: sodium chloride; OD: optical density; orf: open reading frame; PCR: polymerase chain reaction; RISF: TPase/retroviral integrase superfamily; RSV: Rous sarcoma virus; SC: synaptic complex; Tpase: transposase; Tris-Cl: tris(hydroxymethyl)amino methane; Vhr: volt hour.

## Competing interests

The authors declare that they have no competing interests.

## Authors' contributions

PTU created the fusion construct, carried out the overexpression and protein purification experiments, the secondary structure analysis and *in silico *determination of the amino acid substitutions in the mutant strains. SA carried out all cloning experiments involving the creation of plasmids with the *orfAB *mutations. RS performed the PCR and PCR-based mutagenesis experiments. JA carried out all of the *lacZ *papillation experiments. LAL designed the study and provided facilities and funding. LAL and MA wrote the manuscript. All authors have read and approved the final manuscript.
